# Distinct triterpene synthases in the laticifers of *Euphorbia lathyris*

**DOI:** 10.1038/s41598-019-40905-y

**Published:** 2019-03-18

**Authors:** Edith Forestier, Carmen Romero-Segura, Irini Pateraki, Emilio Centeno, Vincent Compagnon, Myriam Preiss, Anne Berna, Albert Boronat, Thomas J. Bach, Sylvain Darnet, Hubert Schaller

**Affiliations:** 1 Plant Isoprenoid Biology team, Institut de Biologie Moléculaire des Plantes, UPR2357 du CNRS, Université de Strasbourg, 12 rue du Général Zimmer, Strasbourg cedex, 67084 France; 2 Center for Research in Agricultural Genomics (CSIC-IRTA-UAB-UB), Bellaterra, Spain; 3 Department of Biochemistry and Molecular Biomedicine, Faculty of Biology, University of Barcelona, 08028 Barcelona, Spain; 4 Instituto de Ciências Biológicas, Universidade Federal do Pará, Pará, Brazil

## Abstract

*Euphorbia lathyris* was proposed about fifty years ago as a potential agroenergetic crop. The tremendous amounts of triterpenes present in its latex has driven investigations for transforming this particular biological fluid into an industrial hydrocarbon source. The huge accumulation of terpenes in the latex of many plant species represent a challenging question regarding cellular homeostasis. In fact, the enzymes, the mechanisms and the controllers that tune the amount of products accumulated in specialized compartments (to fulfill ecological roles) or deposited at important sites (as essential factors) are not known. Here, we have isolated oxidosqualene cyclases highly expressed in the latex of *Euphorbia lathyris*. This triterpene biosynthetic machinery is made of distinct paralogous enzymes responsible for the massive accumulation of steroidal and non-steroidal tetracyclic triterpenes. More than eighty years after the isolation of butyrospermol from shea butter (Heilbronn IM, Moffet GL, and Spring FS *J*. *Chem*. *Soc*. 1934, 1583), a butyrospermol synthase is characterized in this work using yeast and *in folia* heterologous expression assays.

## Introduction

*Euphorbia lathyris* is a herbaceous plant native to the Mediterranean area and widespread in temperate regions. Its vascular tissues show a dense network of accompanying laticifers^1^. This is also the case of all *Euphorbia* species and other plants from the Euphorbiaceae family, including the popular rubber tree *Hevea brasiliensis*^2^, and species in several plant families like the Moraceae, Apocynaceae, and Papaveraceae^3,4^. A major trait of these plants is the massive accumulation of specialized metabolites in the laticifers, like for instance the tremendous amounts of the alkaloids morphine and codeine found in the laticifers of opium poppy^5,6^. Laticifers in many Euphorbiaceae grow and form anastomoses throughout the plant from the embryo stage^7,8^. The cytoplasm of these coenocytic cells is a milky fluid called latex that contains heavy loads of isoprenoids: *cis*-1,4-polyisoprene otherwise known as natural rubber in *H*. *brasiliensis*, and very often high concentrations of diterpenes or triterpenes in *Euphorbia* species^9,10^. The biosynthetic pathway of *E*. *lathyris* seed oil diterpenoids (called euphorbia factors) has been fully described from the casbene precursor to the bioactive macrocyclic diterpene derivatives such as ingenol^11^.

The genus *Euphorbia*, comprising almost 2000 species, has been in the focus of chemotaxonomic studies from the early sixties. This was mostly inspired by the challenge of establishing phylogenetic relationships based on architectural, morphological, anatomical, physiological and chemical traits available at that time. Whether the observed chemical diversification acts as a driving force in morphological evolution remains unclear so far. At least the coincidental occurrence of a very specific triterpene signature with a particular set of morphological traits was observed in some cases, despite of the complex geographical distribution of *Euphorbia* species. These pioneering phytochemical surveys have revealed the triterpene (C_30_H_50_O) skeletal diversity in the latex of *Euphorbia* species^12^. *Euphorbia lathyris* contains mostly lanostane (lanosterol, cycloartenol and their C24-methylated derivatives), euphane (butyrospermol, euphol), and hopane (hopenol-B) derivatives in its latex^13,14^. The presence of a triterpene of the bacterial-type, namely, hopenol-B, was also found in *Euphorbia supina*^15^. In reality, the chemical composition of the latex of *Euphorbia lathyris* is much more diverse^16^, and several studies have reported the presence of other substances than terpenoids like for instance L-dopa^17^. Preceding the more recent interest in *Euphorbia spp* for pharmacologically active compounds including terpenoids^11^, *Euphorbia lathyris* was proposed about fifty years ago as a potential agroenergetic crop by Melvin Calvin^18,19^. In fact, *Euphorbia lathyris* latex is heavily loaded with extractable organics mainly consisting of fermentable sugars and huge amounts of triterpenes, which may represent up to 50% of the latex dry weight^20^. Such energy-rich triterpenoids can be extracted and converted into a gasoline-type biofuel in oil refinery units of pre-industrial scale^21^.

Polycyclic triterpenes are synthesized through the action of oxidosqualene cyclases (OSCs), also called triterpene synthases, which convert the substrate 2,3-oxidoqualene into one or several products belonging to one or more groups of compounds having a defined triterpene backbone. Nearly two hundred skeletons have been reported for monocyclic, bicyclic, tricyclic, tetracyclic, and pentacyclic triterpenes^22^. The mechanism of the corresponding enzymatic cyclizations was already described in a seminal paper by Eschenmoser *et al*.^23^ and since then studied extensively by means of enzymology, molecular cloning of the catalysts, site-directed mutagenesis and structural studies contributed by several groups, including those tackling the cognate squalene cyclization into hopane triterpenes^24–33^. Achievements regarding plant triterpene synthases have been reviewed in details^34,35^. The conversion of (*3S*)-2,3-oxidosqualene into (poly)cyclic products is initiated by its protonation and then followed by cyclization in a series of cationic intermediates that are typical of an OSC family and corresponding to one or more backbones. The deprotonation and release of the final triterpene by an OSC active site is preceded by a series of rearrangements (1,2-shifts of hydride and methyl groups) that vary in number between the types of OSCs and therefore define the triterpene skeletal diversity mentioned above. To a large extent, this diversity is responsible for the wealth of pentacyclic compounds known today, including compounds as baccharane, lupane, oleanane, ursane, taraxane and friedelane series, all being cyclized from the (*3S*)-2,3-oxidosqualene substrate in chair-chair-chair conformation via the dammarenyl cation prior to further rearrangements^11,24^. The wide distribution of β-amyrin among those pentacyclic triterpenes promoted its designation as the ‘most popular triterpene” by Ebizuka and coworkers^36,37^. Such a ubiquitous nature of β-amyrin in plants is most probably linked to one or more functions of this compound in some physiological or biological processes. In fact, the implication of β-amyrin in epidermal cell fate decision to differentiate first into trichoblasts and then into root hair cells in *Avena strigosa* was proposed recently: a superhairy root phenotype of a saponin-deficient mutant was associated with a dramatic increase in β-amyrin^38^.

**Figure 1:**
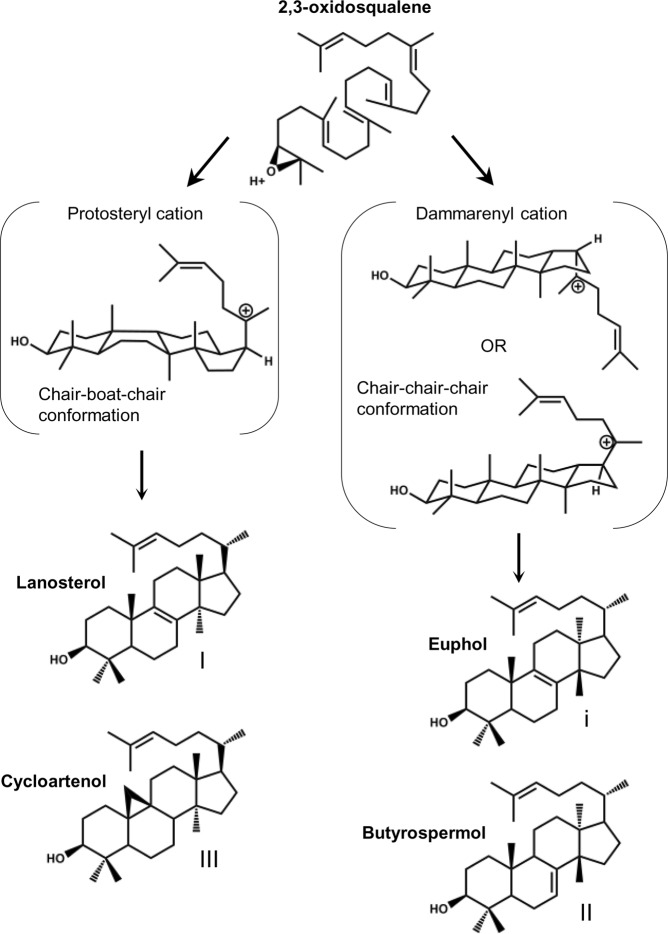
Cyclization of 2,3-oxidosqualene into tetracyclic triterpenes. Lanosterol or cycloartenol synthases cyclize the protonated 2,3-oxidosqualene in a chair-boat-chair conformation via a protosteryl cation, whereas butyrospermol or euphol synthases cyclize 2,3-oxidosqualene into butyrospermol or euphol via epimeric 17α- and 17β-dammarenyl cations^22,24^.

The production of tetracyclic triterpenes in *Euphorbia lathyris* follows two biosynthetic routes. The substrate 2,3-oxidosqualene in chair-boat-chair conformation is cyclized into cycloartenol or lanosterol. The protosteryl cationic reaction intermediate is rearranged into a lanosteryl cation prior the final C-19 or C-8 deprotonations to yield the steroidal tetracyclic triterpene cycloartenol or lanosterol, respectively. Alternatively, the substrate in all-chair conformation is cyclized into the dammarenyl cation, rearranged into the cation euphane prior to the final deprotonation at C-7 or at C-8, to yield the non-steroidal tetracyclic triterpene butyrospermol or euphol, respectively (Fig. 1). Butyrospermol is the major product of the latter cyclization whereas lanosterol and cycloartenol (and its derivative 24-methylene cycloartanol) are two main products of the former. Lanosterol is generally a rare plant triterpene although a few species have genes encoding true lanosterol synthases just like fungi or mammals, according to the products formed by the corresponding enzymes in heterologous expression systems^39,40^. The presence of a functional lanosterol synthase in these organisms is however dispensable since for instance loss-of-function mutants of *Arabidopsis thaliana* grow and develop normally like wild-type plants^41^. Very few plant species produce specialized metabolites that are derivatives of lanosterol, such as lanosterol oligosaccharides in certain Liliaceae, suggesting possible species-specific functions^42^. Cycloartenol synthases have been detected in all plant genomes sequenced so far and also in some Protista, and many were characterized in functional assays^43^. The mutational spectrum of cycloartenol synthase has been studied in molecular evolution experiments that pointed out some catalytically important residues of the protein. Notably, these mutagenesis experiments have revealed new cycloartenol synthases isoforms that were able to form lanosterol or parkeol (obtained by deprotonation at C-11 of the lanosteryl cation) in addition of cycloartenol^28,44^. This illustrates the flexibility of triterpene synthases, which was also described for several non-steroidal triterpene synthases from *Arabidopsis thaliana* able to generate an array of products when expressed in a yeast strain deficient in its endogenous lanosterol synthase^45^. Interestingly, some of these versatile catalysts like At1g78960/LUP2 from *Arabidopsis thaliana*^46^ or PSM/BAA97559 from *Pisum sativum*^47^ were shown to produce small amounts of butyrospermol among a variety of tetracyclic and pentacyclic triterpenes^34^.

Here, we have characterized the triterpene biosynthetic machinery of the latex of *Euphorbia lathyris*. Whether all triterpenes found in *Euphorbia lathyris* are produced by a nonspecific versatile catalyst or by distinct enzymes is unraveled here. The analysis of the latex transcriptome revealed a complete isoprenoid pathway in full agreement with previous data on the bioconversion of acetate or mevalonate into triterpenes by latex fractions^10^. Using yeast and *in folia* heterologous expression approaches we have shown that distinct triterpene synthases are responsible for the accumulation of tetracyclic triterpenes in the latex.

## Results and Discussion

### A triterpene “*lathyris*” signature

Triterpene and sterol profiling of *Euphorbia lathyris* revealed a very rich and diverse content of free or acylated derivatives, and significant amounts of sitosterol glucoside (Fig. S1). Striking differences in triterpene profiles were associated with plant developmental stages or organ specificity (Fig. S2, Table S1). Such variability of the triterpenoid constituents of *E*. *lathyris* seedlings was already reported in the case of photomorphogenesis versus skotomorphogenesis^48,49^. This contrasts with the remarkably stable composition of the latex triterpene profile. It shows five prominent compounds just as a specific “*lathyris*” signature, and a group of minor ones, of which euphol belongs to the dammarane series (Fig. S3, Table S1). The only qualitative variation in this profile was the conditional presence of 24-methylene lanosterol, a compound that peaked up depending on the growth conditions, in a greenhouse or open-field conditions (Fig. S3). More striking was the quantitative variation of the “*lathyris*” triterpene signature that increased up to three-fold when plants were exposed to elevated temperatures (Fig. S4). Such an increase in isoprenoid biosynthesis upon heat exposure has been associated with thermal tolerance in other plants^50^.

### Latex-specific triterpene synthases

**Figure 2:**
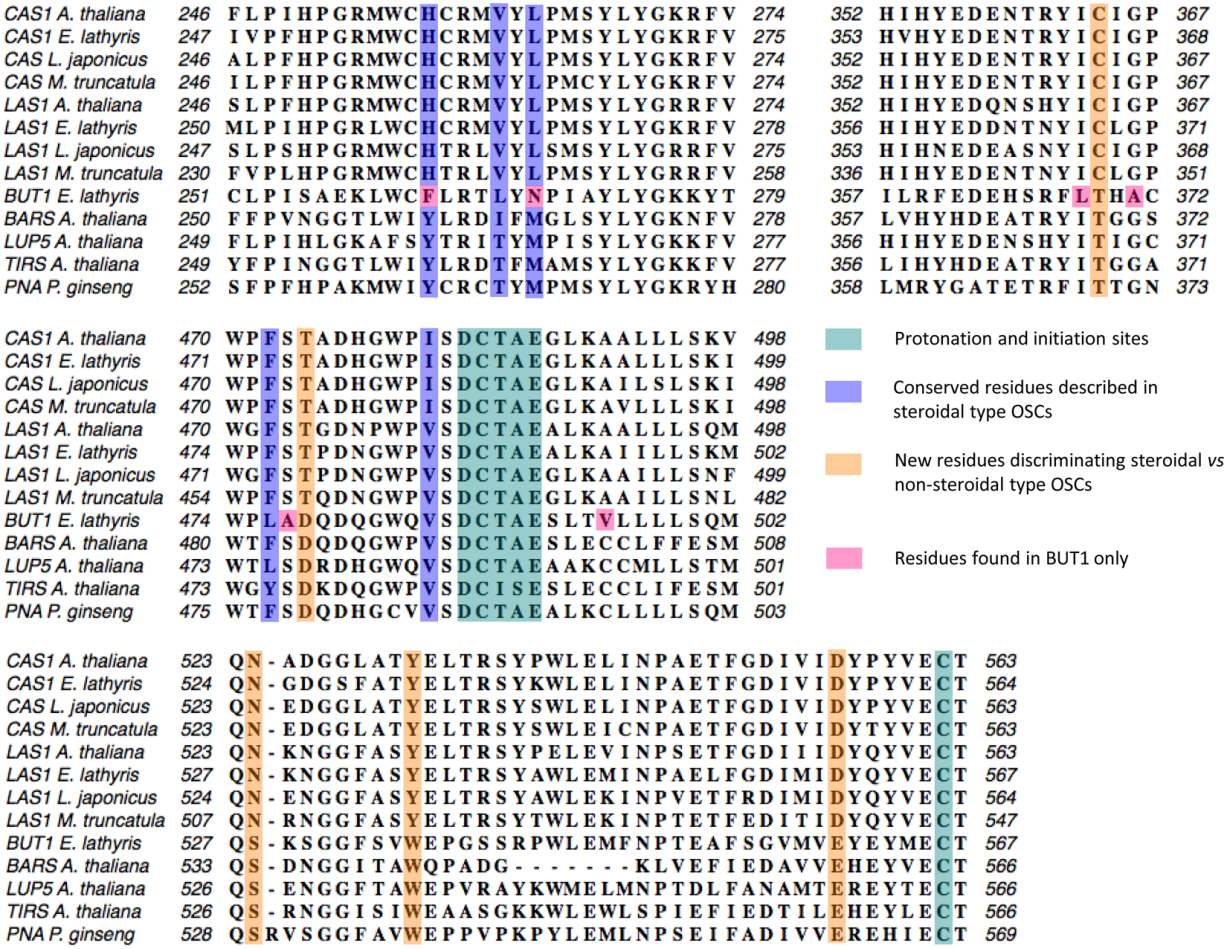
Alignment of higher plant OSCs implied in tetracyclic triterpene synthesis. Multiple sequence alignments were performed with MacVector software using the ClustalW program and BLOSUM 62 matrix. Important conserved or variable amino acid residues and motifs are shown. Lanosterol synthases (LAS1) accessions are: *A*. *thaliana*, At3g45130^39,40^; *L*. *japonicus*, AB244671.1^40^; *M*. *truncatula*, XP_013453255.1^73^; *E*. *lathyris*, this work; Cycloartenol synthases (CAS1) accessions are: *A*. *thaliana*, At2g07050^25^; *L*. *japonicus*, BAE53431 (Sawai *et al*., 2004); *M*. *truncatula*, XP_003610947^73^; *E*. *lathyris*, this work. Non-steroidal tetracyclic triterpene synthases accessions are: *E*. *lathyris*, BUT1, this work; *A*. *thaliana* baruol synthase BARS, At4g15370^45^; *A*. *thaliana* tirucalledienol synthase LUP5, At1g66960^74^; *A*. *thaliana* tirucalladienol TIRS/PEN3, At5g36150^52^; *P*. *ginseng* damarenediol-II synthase/PNA^54^, AB265170.

**Figure 3:**
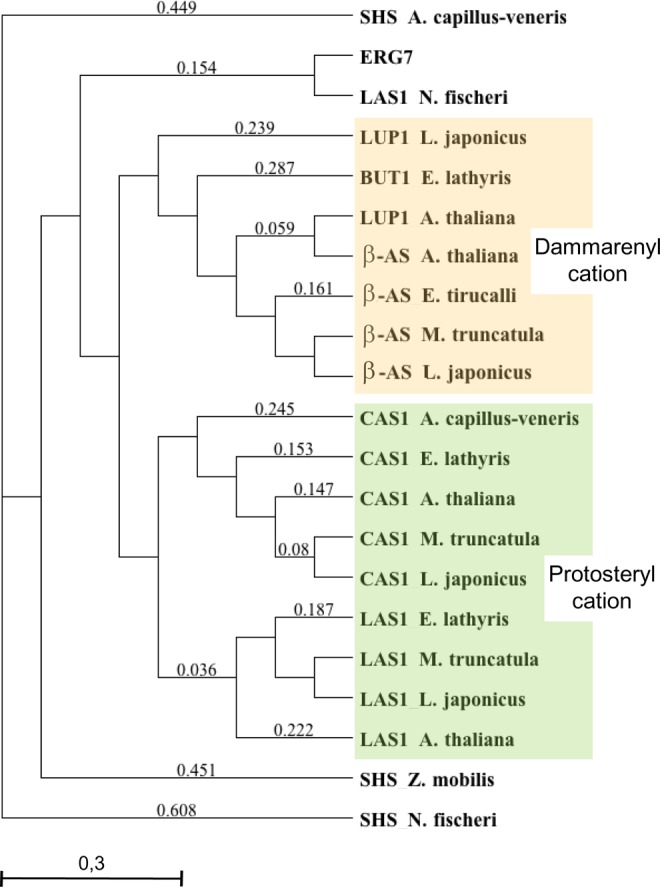
Maximum likelihood phylogenetic tree based on amino acid sequences of squalene-hopene synthase (SHS) and OSCs. Squalene-hopane cyclase accessions: *Neosartorya fischeri* (DS027688.1), *Zymomonas mobilis* (X73561), *Adiantum capillus-veneris* (AB368376). Lanosterol synthase accessions: *Saccaromyces cerevisiae ERG7* (U04841), *Neosartorya fischeri* (DS027688.1), higher plant sequences as in Fig. 2. Cycloartenol synthase accessions as in Fig. 2. Dammarane-type triterpene synthases: *Lotus japonicus* LUP1 (AB181245), *Euphorbia lathyris* BUT1 (this work), *Arabidopsis thaliana* LUP1 At1g78970 (NM_106546)^15,75–82^; β-amyrin synthases: *Arabidopsis thaliana* At1g78950 (AB374428), *Euphorbia tirucalli* (AB206469), *Medicago truncatula* (CAD23247), *Lotus japonicus* (AAO33580).

The transcriptome of *Euphorbia lathyris* latex generated in this study was searched for OSCs using chosen sequences (listed in methods section) and available transcriptome data from *E*. *lathyris* seeds^11^ and *Euphorbia tirucalli*^51^. This exhaustive database search resulted in the identification of three cDNAs named *El*LAS1, *El*CAS1, and *El*BUT1 (Figs S5 and S6). Protein sequence alignment with triterpene synthases from plants, yeast and bacteria pointed out in each of the three sequences the DCTAE motif and the C562 residue being generally implied in the initial substrate protonation (Fig. 2). In addition, one of the OSCs had an I481 (*A*. *thaliana* LAS1 numbering) whereas the other two had a V481 (Fig. 2). Cycloartenol synthases are specified by an I481 that seems strictly required for the 9β,19-cyclopropyl ring formation, whereas lanosterol synthases and other triterpene synthases like tirucalladienol synthases from *A*. *thaliana* named TIRS and LUP5 have a Val residue at the same position^52^. Phylogenetic relationships between additional triterpene synthases from plants, yeast and bacteria gave valuable information on the putative functions of the three *E*. *lathyris* OSCs. Two of these OSCs grouped together with the lanosterol and cycloartenol synthases (LAS1 and CAS1) that convert the protosteryl cation into steroidal triterpenes (Fig. 3). The third sequence belongs to a cluster of triterpene synthases that all convert the dammarenyl cation in non-steroidal products; this sequence was therefore distantly related to the previous ones and finally named BUT1 (Fig. 3). Molecular evolution and site-directed mutagenesis of OSCs have been reported by several authors to identify critical residues for successful 2,3-oxidosqualene cyclization^28,45^. Furthermore, a comprehensive, phylogenetic systematic analysis based on the crystal structure of the *Alicyclobacillus acidocaldarius* squalene hopene cyclase^32^ and the *Homo sapiens* lanosterol synthase^33^ and a compilation of 639 other cyclases in a triterpene cyclase engineering database gave a model to help predicting a function of a triterpene synthase based on sequence conservation analysis^53^. Important substrate-interacting residues and second sphere residues were reported in addition to previous structure-function analyses. At present, only a few OSCs that produce polycyclic triterpenes from the dammarenyl cation have been isolated: the dammarenediol II synthase from *Panax ginseng*^54^ and the tirucalladienol synthases *At*TIRS and *At*LUP5 from *Arabidopsis thaliana*^52^. Some novel features of the OSCs protein sequences may be drawn here. The steroidal triterpene synthases CAS1 and LAS1 had a conserved H257, V261, L263 whereas the dammarane-type enzymes display an Y257, T261, M263 except BUT1 that has a F257, L261, N263 (*At*LAS1 numbering). Some other residues clearly discriminated steroidal versus non-steroidal dammarane-type OSCs (C364T, T474D, N524S, Y532W, D556E) and strinkingly, OSCs known to produce butyrospermol when expressed in yeast (*At*BARS, *At*TIRS; Lodeiro *et al*., 2007; Morlacchi *et al*., 2009) had a Q477, which is also the case of *El*BUT1 (Fig. 2). Theses sequence variations may be interesting to challenge further a clear-cut identification of dammarane-type synthases. Position 474 of a β-amyrin synthase from *Euphorbia tirucalli* was shown as essential for proper folding of the substrate and completion of the cyclization reaction^55,56^. Residues H257 and Y532 that are typically found in cycloartenol synthases and lanosterol synthases are suggested to play a role in the tetracyclic cation generation and final deprotonation at C-19^57^. A further sequence comparison with the non-steroidal tetracyclic triterpene synthases indicate that dammarane-type OSCs have instead non-polar amino acid residues at these positions (tryptophane and phenylalanine, respectively). Based on the crystal structure of the human oxidosqualene cyclase^33^, F472 and Y532 (AtLAS1 numbering) are possibly involved in the stabilization of the intermediate tertiary cation after A-ring and B-ring formation, and C562 could act as hydrogen-bonding partner with D483 of the DCTAE motif^39^. There is currently a lack of sequence information about non-steroidal tetracyclic triterpene synthases to figure out whether one single amino acid residue could discriminate C-7 and C-8 proton abstractions, stabilization of the euphane cation and production of butyrospermol (in the Δ^7^ series) or euphol (in the Δ^8^ series) like it holds true for steroidal tetracyclic triterpenes that differ by the single I481V mutation to form lanosterol or cycloartenol by deprotonation at C-9 or C-19 of the lanostane cation.

### Functional analysis of the latex triterpene synthases

**Figure 4:**
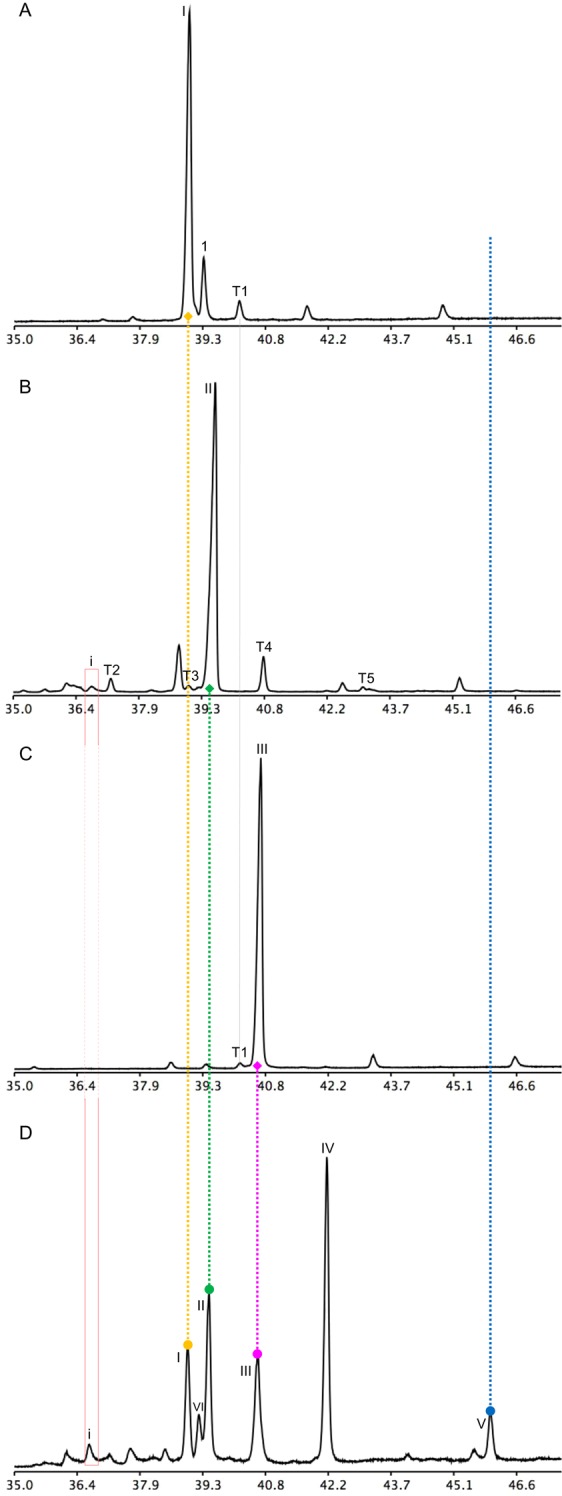
Chromatograms (TIC, GC-MS) showing the triterpene profiles of the yeast *erg7* transformed with triterpene synthases from *Euphorbia lathyris*. (**A**) *erg7::ElLAS1*; (**B**) *erg7::ElBUT1*; (**C**) *erg7::ElCAS1*; (**D**) latex triterpenes shown as standard references. Dashed lines show the alignment of products of interest with same retention times. Nomenclature: I, lanosterol; II, butyrospermol; III, cycloartenol; IV, 24-methylene cycloartanol; V, hopenol-B; VI, 24-methylene lanosterol; nomenclature of minor products is given in Table S1. Peaks that are not numbered are not terpenoids.

The triterpene synthases cloned from the latex total RNA were expressed in the yeast *erg7* (lanosterol synthase deficient, ergosterol auxotroph mutant, Fig. S7). Transformation of the yeast *erg7* with *ElLAS1* yielded lanosterol (Fig. 4A) and its downstream metabolite 4,4-dimethylzymosterol formed by the residual ergosterol biosynthetic machinery present in *erg7*. This sterol intermediate enters the ergosterol pathway and is converted into ergosterol. This is proven by the autotrophic growth of *erg7*:: *ElLAS1* (data not shown) and the strong isotopic enrichment of sterols from yeast grown solely on [1-^13^C]-galactose (converted into glucose by the yeast epimerase) as a carbon source in the medium (Fig. S8). Transformation of *erg7* with the dammarane-type triterpene synthase allowed the detection of butyrospermol as the prominent compound (Fig. 4B). A very tiny peak of euphol was detected in the triterpene fraction of *erg7::BUT1* yeasts. Large-scale cultures of this strain were produced and extracted to search for additional compounds, especially the missing hopenol-B in the products formed by the three *Euphorbia lathyris* OSCs. At this stage it cannot be excluded that hopenol-B might be cyclized from the dammarenyl cation upon D-ring expansion and E-ring cyclization to form a hopyl cation, and not from the direct cyclization of 2,3-oxidosqualene into a hopyl cation^22^. The triterpene fractions analyzed in GC-FID and GC-MS did not show any traces of hopenol-B. Euphol was detected as shown in Fig. 4B and also a pentacyclic triterpene possibly of the lupane-type (according to the mass spectrum, Table S1) having however a very different mobility than hopenol-B. *ElCAS1* produced cycloartenol (Fig. 4C) as the main product when expressed in *erg7* as well as small amounts of 9β,19-cyclopropylsterol derivatives detected in the sterol fraction (not shown here) as proven for other plant CAS1s^58^.

**Figure 5:**
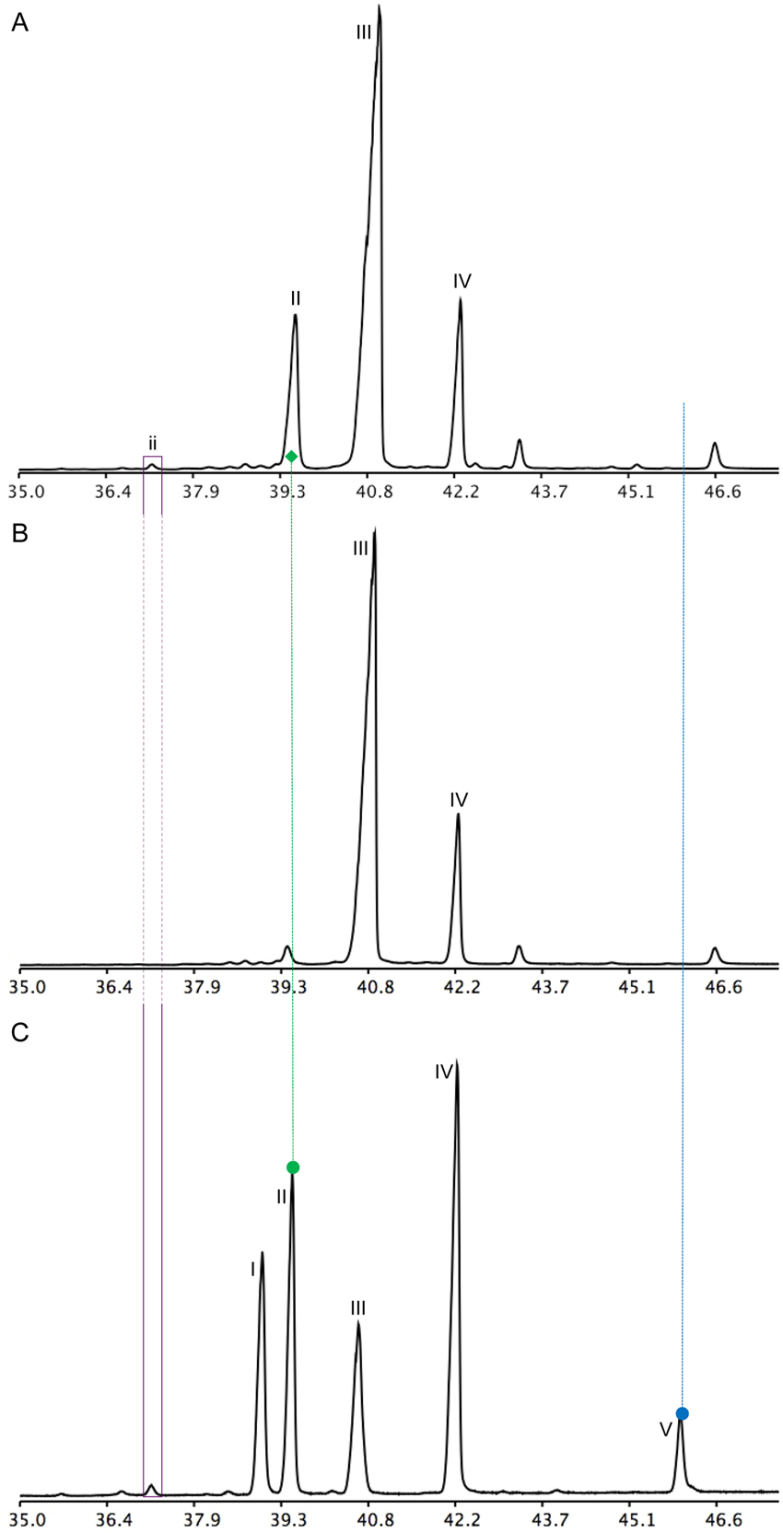
Chromatograms (TIC, GC-MS) displaying the triterpene profile of *Nicotiana benthamiana* leaf tissues after *in folia* agroinfiltration of *ElBUT1*. (**A**) TLC-purified triterpene fraction of leaves infiltrated with T-DNA constructs driving the expression of *HMGR* and *BUT1*. (**B**) TLC-purified triterpene fraction of leaves infiltrated with a T-DNA construct driving the expression of *HMGR* (control assay). (**C**) Latex triterpenes shown as a reference. Butyrospermol (II) is absent from wild-type *N*. *benthamiana* leaves as well as from leaves transformed with HMGR, only therefore the wild-type TIC analysis is not shown. The dashed line indicates the correspondence of butyrospermol produced in *N*. *benthamiana* and the latex standard. Nomenclature: I, lanosterol; II, butyrospermol; III, cycloartenol; IV, 24-methylene cycloartanol; V, hopenol-B; nomenclature of minor products is given in Table S1. Peaks that are not numbered are not terpenoids.

A clear-cut validation of the nature of the new OSC catalyst was obtained from the *in folia* expression of *ElBUT1* using the *Nicotiana benthamiana* (*Niben*) leaf infiltration assay^59^. To increase the biosynthetic flux in the mevalonate pathway leading from acyl-CoA to 2,3-oxidosqualene, a tobacco cDNA encoding a truncated, deregulated isoform of the enzyme 3-hydroxy-3-methylglutaryl-coenzyme A reductase (HMGR) was co-expressed with *ElBUT1*. Butyrospermol and the endogenous cycloartenol and 24-methylene cycloartanol were the most abundant triterpenes of such leaf extracts (Fig. 5A), whereas only cycloartenol and its derivatives were found in controls (Fig. 5B). Minor triterpenes were detected in the latex, in *erg7*::*BUT1*, and in *Niben*::*BUT1* but not in relevant controls, strongly suggesting that they are products of BUT1 (Fig. 5B). The major triterpenes of leaf extracts and of *Euphorbia lathyris* latex were quantified (Fig. S9), purified by silver nitrate thin layer chromatography, then subjected to NMR analysis as their acetate derivatives. The structure of butyrospermol produced by BUT1 in the plant leaf cellular context (*Niben*) was identical to that of latex butyrospermol (Fig. S10).

Altogether, the bioassays implemented here showed that the main tetracyclic triterpenes from the latex of *E*. *lathyris* are produced by distinct lanosterol and cycloartenol synthases and by a novel OCS named BUT1 responsible for the cyclization of 2,3-oxidosqualene into butyrospermol.

### Accuracy of the steroidal triterpene synthases of *Euphorbia lathyris*

Our results revealed that at least three OSCs are required to provide the short compendium of latex triterpenes. Each of these enzymes was able to catalyze the synthesis, upon expression in yeast and *in folia*, of a single product, or of an extremely narrow spectrum of compounds (in the case of *El*BUT1). No versatile OSCs such as for instance those capable of converting 2,3-oxidosqualene into cycloartenol, lanosterol, and parkeol^57^ were identified in this study. *El*BUT1 is a novel type of enzyme responsible for the production of butyrospermol, being by far the most prominent product in addition to very minute quantities of other triterpene products (especially, euphol, *vide infra*). This enzyme is distinct from the *A*. *thaliana* tirucalladienol synthases, which use the epimeric 17α-dammarenyl cation to form tirucallane triterpenes^52^. As such, a set of non-steroidal tetracyclic triterpene synthases represent valuable adds to the growing repositories of OSCs (e.g. the Triterpene Cyclase Engeneering Database^53^) for a further deciphering effort on the mechanism of dammarane triterpene formation (17α- or 17β-dammarenyl cations, which are rearranged into butyrospermol (20*R* stereoisomer) or into tirucallol and tirucalladienol (20*S* stereoisomer)).

The “*lathyris* triterpene signature” is established by a set of highly specific OSCs: BUT1, CAS1 and LAS1. OSC specificity or accuracy was defined previously by the ratio of the primary product P_1_ to the second most abundant product P_2_ (or to the sum of all products P_i_) formed by a given catalyst when expressed in a recombinant yeast from which a biosynthetically active homogenate was able to convert milligram amounts of 2,3-oxidosqualene into cyclic compounds^45^. Values of P_1_/ΣP_i_ were 0.95 (LAS1), 0.99 (CAS1), and 0.87 (BUT1 in yeast) or 0.98 (BUT1 in *Nicotiana benthamiana*) (Table S2). These values set the *E*. *lathyris* OSCs CAS1 and LAS1 in the group of the highly accurate OSCs^45^ whereas BUT1 is as highly accurate as the latter (Table S2). The rationale of product accuracy values relies on the intricate and prodigiously rich conversion of 2,3-oxidosqualene by the *Arabidopsis thaliana* BARS1 (an OSC named baruol synthase because the most abundant product found in yeast was baruol) to mono-, bi-, tri-, tetra-, and penta- cyclic triterpenes of which butyrospermol accounted for 1% of the total formed products^45^. For instance, the *A*. *thaliana* LUP1 and LUP2 are responsible for the formation of similar amounts of lupane, oleanane, ursane, and taraxane derivatives, and therefore classified as “multifunctional” (although *sensu stricto* their only known function is that of an OSC, which is a biochemical function *per se*). The most accurate cyclases are the steroidal triterpene synthases, which are essential for viability, namely CAS1 in plants^60^, and LAS1 in fungi and mammals.

### Laticifers as a triterpene factory

The presence of both CAS1 and LAS1 in laticifers raises several questions. In fact, the exact nature of sterol requirements in laticifers is not known. A complete set of transcripts encoding sterol biosynthetic enzymes being implied in the conversion of 2,3-oxidosqualene into sitosterol has been identified in the latex transcriptome (Table S3). Laticifers contain sitosterol at levels inferior to the overload of cycloartenol (and cycloartenol esters) but certainly sufficient to maintain cell homeostasis, which contributes to make *E*. *lathyris* a triterpene-rich species. The identification of the cellular machinery that regulates the orchestration between the pool of cycloartenol used for the synthesis of sterols or the one that accumulates in the latex represents a challenging research topic. The laticifers form a tubing network of single cells (coenocytes) that is unique because of the elongation of laticifer cell initials without formation of a phragmoplasts and subsequent *bona fide* cell division (only organelles and nuclei divide). Laticifers formation occurs as soon as the embryo starts to develop and forms the typical non-articulated laticifer networks of *E*. *lathyris*^61^. The non-photosynthetic latex cells and their extraordinary elongation capacity are reminiscent of the pollen tube growth in angiosperm that displays a very specific sterol biosynthetic capacity^62^. In the same vein, a minimal sterol pathway is known in *Gemmata obscuriglobulis*, a bacteria that converts squalene into 2,3-oxidosqualene, then to lanosterol and parkeol as final isomeric products, recapitulating the pathway to a single post-oxidosqualene biosynthetic gene^63^. Laticifers express in their transcriptome sterol-C24-methyltransferases^64^ of the SMT1 type (Table S3) to produce 24-methylene derivatives of cycloartenol and lanosterol, making the laticifer-specific lanostane pathways a juxtaposition of two simple pathways, both encompassing two post-oxidosqualene biosynthetic genes. The physiological relevance of these particularities is not known. Furthermore, the metabolic link between a massive cycloartenol/lanosterol deposition and a possible phytosterol biosynthesis diverted from those pools of committed precursors, at present solely based on transcripts identification (Table S3), is not known either. Laticifers are apparently dispensable for growth at least in standard greenhouse conditions as proven by the *pil* (*poor-in-latex*) mutants of *E*. *lathyris* that lack detectable laticifers and consequently the ‘*lathyris* triterpene signature’^61^. Laticifers still remain intriguing regarding their metabolic interference with surrounding plant cells and possibly other ones from their microbiota^65^. The identification of the currently unknown entities producing hopenol-B is a next challenge to deepen the understanding of diversity and evolution of OSCs in plant cells^66^.

Finally, triterpenes represent a valuable multipurpose renewable phytochemical resource for the energy, and (most importantly), for the pharmaceutical, advanced materials, and cosmetic industrial sectors. Hence, our study like many others of the same type has a great significance in modern chemistry including functional nanochemistry towards a sustainable society^67^.

## Material and Methods

### Plant material

*Euphorbia lathyris* L. plants were grown in a growth chamber under a long-day light regime at 24 °C during the light phase (16 h) and 20 °C during the dark phase (8 h). Seeds were germinated in standard garden soil for one month then seedlings of 20–30 cm in height were transferred into individual pots. Latex samples were collected through razor-blade transversal sections of stems starting from the apices to bottoms and every 10 cm to take advantage of the pressure in laticifers to drain the tissues as much as possible. *Nicotiana benthamiana* Domin. plants were grown in a standard soil mixture (Archut Fruhstorfer Erde®) in a growth chamber maintained under a long-day light regime (16 h light, 120 μmol photons m^−2^ sec^−1^).

### Generation of latex transcriptome database

Total RNA was extracted from 300 μL of fresh latex sap collected from 3-month-old greenhouse grown *Euphorbia lathyris* plants. Plant stems were sectioned in apical, middle, and bottom parts in order to assist the outflow of the latex. In brief, extraction of total RNA was achieved by phenol/chloroform phase extraction followed by a LiCl precipitation step according to standard procedures. The final cleanup of samples was performed with the RNeasy Plant Mini Kit (Qiagen). Quality control of the latex RNAs was performed with the Agilent 2100 Bioanalyzer. For the generation of latex transcriptome database using Next Generation Sequencing technologies, total RNA from latex (2.5 μg total RNA from apical stem sections and 2.5 μg total RNA from middle stem sections) was used to prepare libraries for paired-end Illumina Genome Analyzer II (Solexa) sequencing according to the manufacturer instructions. A total of 44 million reads of an average size of 114 bp provided a sequence of about 5 Gb. The transcriptome assembly using ABySS and Velvet software predicted 23395 contigs of 0.8 kb length on average. The annotation of the transcriptome was done with Gene Ontology tools.

### Identification and isolation of *Euphorbia lathyris* oxidosqualene cyclases (OSCs)

The transcriptome sequence of *Euphorbia lathyris* latex was used to search for sequences encoding OSCs after appropriate filtering of contigs. The following queries identified by their GenBank references were considered for this search. Triterpene synthases: AB206470 and AB206469, a putative 2,3-oxidosqualene cyclase and a β-amyrin synthase from *Euphorbia tirucalli*, respectively; HM623871, HM623870, HM623869, and HM623868, a lupeol synthase, a friedelin synthase, a glutinol synthase and a taraxerol synthase, respectively, from *Kalanchoe daigremontiana*. Lanosterol synthase: NM_114382 from *Arabidopsis thaliana*. Cycloartenol synthase: HM623873, ABB 76767, AAC04931, from *Kalanchoe daigremontiana*, *Ricinus communis*, and *Arabidopsis thaliana*, respectively. These target sequences led to the identification of 3 distinct cDNA sequences named *BUT1*, *LAS1*, and *CAS1*, respectively. Polypeptides encoded by these proteins shared 49% (BUT1 vs LAS1), 50% (BUT1 vs CAS1), and 50% (LAS1 vs CAS1) identity, respectively.

### Yeast expression experiments

The cDNAs encoding BUT1, LAS1 and CAS1 from *Euphorbia lathyris* latex were amplified from latex total RNAs and subcloned into a standard cloning vector using the following primers:

BUT1_1.FOR(BamHI): TAT GGATCC ATGTGGAAGCTTGAGGTTGC

BUT1_1.REV(KpnI): TAT GGTACC TTAATCACAATTAATCATATGCTTTCTG

LAS_1.FOR_FL(BglII): TAT AGATCT ATGTGGAAGCTGAAGATATCAG

LAS_1.REV(KpnI): TATGGTACCTTAACTATTATTGTGAGAAGAGAGC

CAS1.FOR(BamHI): TAT GGATCC ATGTGGAGGTTAAAGATTGCTGAGG

CAS1.REV(XbaI): AAT TCTAGA CTTATGAAGCCTGCTGCTTCAGTAC

After sequencing, the cDNAs were subcloned into the pYES2 vector (Thermo Fisher Scientific) into the *Bam*HI (5′) and *Kpn*I (3′) restriction sites.

The pYES2 vector allows galactose-inducible expression of the OSCs upon transformation of the yeast strain GIL77 that carries a null *erg7* allele^36,68^. GIL77 cells were grown in standard yeast culture medium (YPG) supplemented with ergosterol (20 mg. L^−1^) and transformed according to the lithium acetate method^69^. The selection of transformants was performed on a yeast nitrogen base (YNB) medium without amino acids and the galactose induction required the replacement of glucose by galactose in 3-day-old 10 mL cultures of *erg7::ElOSCs* strains. Yeast growth complementation assays were done in the presence of D-[1-^13^C]-galactose (Omicron Biochemicals, Inc., South Bend, IN, USA). Spotting assays for strains plated on YNB medium in the presence or absence of ergosterol were done for dilutions to the tenth of a starting culture taken at OD_600_ = 0.3.

### *In folia* expression experiments with *Nicotiana benthamiana*

*BUT1* cDNA was amplified from pYES2::BUT1 vector with primers BUT1FOR(SpeI): TAAACTAGTATGTGGAAGCTTGAGGTT and BUT1REV(XhoI)TATCTCGAG TTAATCACAATTAATCATATG and subcloned into pBASTA-A4 in-house vector. The pBASTA-A4::BUT1 and the pBI121.1::CD-HMGR^70^ vectors were mobilized into *Agrobacterium tumefaciens* LBA4404 carrying the pTi plasmid pAL4404^71^. For *Nicotiana benthamiana* leaf infiltration of *Agrobacterium*, single colonies were amplified in 2.5 mL of LB medium containing *ad hoc* antibiotics. After an overnight growth at 28 °C, cells were pelleted by centrifugation at 4000 g (5 min), then washed in sterile water at least 4 times. The clean *Agrobacterium* pellet was resuspended in sterile water at a final OD_600_ of 0.6. An *Agrobacterium* strain LBA4404 carrying the silencing suppressor P19^72^ was grown the same way. A typical agroinfiltration assay^59^ consisted in mixing *Agrobacterium* strains of interest (for instance: CD-HMGR + BUT1 + P19), then injecting the solution with a needleless syringe through the epidermis of the abaxial side of leaves. For triterpene extraction leaves were harvested 4 days after the inoculation.

### Triterpene and sterol analysis

Latex (1 to 100 mg, dry weight), leaf and seedling tissues (100–200 mg, dry weight) or freeze-dried yeast pellet samples were homogenized with an Ultra-Turrax blender and saponified in 10–25 mL of 6% KOH in MeOH for 2 h at 80 °C. The non-saponifiable compounds were extracted with three volumes of *n*-hexane. The dried residue was submitted to an acetylation reaction in a mixture of 100 μL of toluene, 50 μL of acetic anhydride and 40 μL of pyridine at 70 °C for 1 h. Alternatively, this dried residue was separated on TLC plates (Merck 60F_254_) using dichloromethane as a developing solvent. Two runs of TLC yielded resolved fractions of 4-desmethylsterols at R_f_ = 0.15, 4α-methylsterols at R_f_ = 0.20, and 4,4-dimethylsterols (triterpenes) at R_f_ = 0.35. Commercial cholesterol and lanosterol were used as TLC standards. An additional silver nitrate TLC was performed to refine the separation of *Nicotiana benthamiana* leaf metabolites. Acetylated triterpene samples were plated on TLC plates (Merck 60F_254_) treated with AgNO3 10% in EtOH/H_2_O (3:1, v/v) and chromatographed with one run of cyclohexane/toluene (65 :35, v/v) mixture. Four fractions B1 to B7 were isolated and scrapped off the plate. Steryl acetates or triterpenyl acetates from crude extracts or TLC fractions were recovered in *n*-hexane and analyzed in gas chromatography (GC). The temperature program of the oven of a Varian 3400 CX chromatograph coupled to a flame ionization detector (GC-FID) or of a Agilent 6890 chromatograph coupled to an Agilent 5973 mass selective detector (GC-MS) included a steep ramp from 60 °C to 220 °C (30 °C.min^−1^) then a 2 °C.min^−1^ ramp from 220 °C to 300 °C, followed by a 10 min plateau. Compounds were separated on a DB5 column for GC-FID (Agilent; 30 m long, 0.32 mm i.d., 0.25 mm film thickness; 2 mL.min^−1^ hydrogen as carrier gas) and quantified using lupenyl-3,28-diacetate as an internal standard. Compounds were separated on a HP5-MS column (Agilent; 30 m long, 0.25 mm i.d., 0.25 mm film thickness; 1 mL.min^−1^ He as carrier gas) for GC-MS and identified by their mass spectra. The silver nitrate TLC of triterpene fractions from *Euphorbia lathyris* latex or *Nicotiana benthamiana* leaf material expressing the cDNA encoding *El*BUT1 yielded the following pure compounds butyrospermyl acetate (B5, R_f_ = 0.29), cycloartenyl acetate (B6, R_f_ = 0.41), lanosteryl acetate (B7, R_f_ = 0.44). A fraction B4 at R_f_ = 0.24 contained 24-methylene cycloartanyl acetate and hopenyl-B acetate. Milligram amounts of pure butyrospermyl acetate from latex and from *Nicotiana benthamiana* were subjected to NMR analysis. ^1^H NMR spectra were recorded in CDCl_3_ with a Bruker Avance 500 instrument. For quantitative and comparative analysis of a triterpene GC profile, biological triplicates allowed the calculation of standard deviations from the mean. Statistics when relevant provided significant different quantitative value (P < 0.05, F-test, T-test, one-way ANOVA).

### Accession numbers

The cDNA sequences encoding *Euphorbia lathyris* LAS1, CAS1 and BUT1 are deposited in the NCBI GenBank under the accessions MH215229, MH215230, and MH2152, respectively.

The transcriptome data sets of *Euphorbia lathyris* generated within the frame of the KBBE project EULAFUEL were deposited at NCBI under SRR7007160, SRR7007159, SRR7007158, SRR7007157, SRR7007156 accession numbers in SRA-NCBI database, BioProject PRJNA450173, Biosample SAMN08932125, Accession GGMG00000000. The transcriptome assembly (TSA) from *Euphorbia lathyris* seeds^11^ BioProject PRJNA282739, BioSample SAMN03576651 was deposited at DDBJ/EMBL/GenBank under the accession GGMH00000000.

Queries and data requests regarding transcriptome data are available from the corresponding author upon request.

## Supplementary information

Distinct triterpene synthases in the laticifers of Euphorbia lathyris.

Table S1

Table S2

Table S3
