# Supplementary material for: Distinct triterpene synthases in the laticifers of Euphorbia lathyris
Source: Sci Rep. 2019 Mar 18;9:4840. doi: 10.1038/s41598-019-40905-y (PMC6423090; doi:10.1038/s41598-019-40905-y)
Supplement: Supplementary file 1 — Distinct triterpene synthases in the laticifers of Euphorbia lathyris. [file 41598_2019_40905_MOESM1_ESM.pdf]

Distinct triterpene synthases in the laticifers of *Euphorbia lathyris.*

Edith Forestier[1], Carmen Romero-Segura[2], Irini Pateraki[2], Emilio Centeno[2], Vincent Compagnon[1], Myriam Preiss[1], Anne Berna[1], Albert Boronat[2], Thomas J. Bach[1], Sylvain Darnet[3], Hubert Schaller[1*]

[1]Plant Isoprenoid Biology team, Institut de Biologie Moléculaire des Plantes, UPR2357 du CNRS, Université de Strasbourg, 12 rue du Général Zimmer, Strasbourg cedex 67084, France

[2]Center for Research in Agricultural Genomics (CSIC-IRTA-UAB-UB), Bellaterra, Spain; Department of Biochemistry and Molecular Biomedicine, Faculty of Biology, University of Barcelona, 08028 Barcelona, Spain

[3]Instituto de Ciências Biológicas, Universidade Federal do Pará, Brazil

***Corresponding author**: Hubert Schaller
hubert.schaller@ibmp-cnrs.unistra.fr

Legends for supplemental figures

Figure S1. 2,3-oxidosqualene-derived groups of metabolites in above-ground tissues of *Euphorbia lathyris*. Amount of triterpenes is in µg/g of dry matter (n=3). Data represent the averages of three biological replicates; error bars indicate the standard deviation from the mean. The number of stars above the bars specifies a significant different quantitative value ($P < 0.05$, F-test and T-test). Each color represents an unrelated statistical group.

Figure S2. Age-related sterol and triterpene GC-MS profiling of *Euphorbia lathyris*. A, total ion chromatogram (TIC) of sterol and triterpenes in the unsaponifiable fraction from 3 week-old seedlings. **B**, triterpene fraction from the unsaponifiable fraction of 3 month-old leaves. **Nomenclature:** I, lanosterol; II, butyrospermol; III, cycloartenol; IV, 24-methylene cycloartanol; V, hopenol-B; VI, 24-methylene lanosterol; 2, campesterol; 3, stigmasterol; 4, taraxerol; 5, sitosterol; 6, isofucosterol; 7, β-amyrin. Mass spectra of triterpenes corresponding

to peaks t1, t2, t3, t4, t5, t6, t7, and t8 are given in Table S1. Differences in retention times of given compounds in panels A and B is due to the implementation of distinct HP5-MS GC columns during the course of the experiments. Peaks that are not numbered do not represent terpenoids.

Figure S3. Triterpenes in the latex of *Euphorbia lathyris*. A, chromatogram (TIC, GC-MS of the triterpene fraction extracted from the unsaponifiable latex of plants grown in the fields in Spain (Madrid area). Major peaks are I, lanosterol; II, butyrospermol ; III, cycloartenol ; IV, 24-methylene cycloartanol ; V, hopenol-B; VI, 24-methylene lanosterol. Minor peak i is euphol (**Table S1**). **B**, chromatogram (TIC, GC-MS) of the triterpene fraction extracted from the unsaponifiable latex of greenhouse-grown plants. Peak ii, unknown triterpene. **C**, Major triterpenes not shown in Figure 1. **D**, amount of triterpenes in the latex shown in B (µg/mg of dry matter, n=3). Peaks that are not numbered do not correspond to terpenoids.

Figure S4. Triterpene accumulation under warm weather conditions. *Euphorbia lathyris* latex was tapped in a glasshouse in spring when plants were grown under natural and progressively warmer and sunny weather within a 3-week timeframe (µg/mg of dry matter, n=3). **A**, I, lanosterol; II, butyrospermol; III, cycloartenol; IV, 24-methylene cycloartanol; V, hopenol-B; VI, 24-methylene lanosterol; **B**, sitosterol glucoside and sitosterol from the same extracts as in A; Data represent the averages of three biological replicates; error bars indicate the standard deviation from the mean. The number of stars above the bars specifies a significant different quantitative value ($P < 0.05$, F-test and T-test). Each color represents an unrelated statistical group.

Figure S5. Transcriptome analysis and OSCs sequence identity in *Euphorbia lathryris*. OSC sequences were retrieved from assembled transcriptomes of *Euphorbia lathyris* latex (this study) and whole seeds [11], and of *Euphorbia tirucalli* [51]. Three OSCs were found in *E. lathyris* latex (contig104_seq4, BUT1; contig848_seq1, LAS1; and contig104_seq1, CAS1). The latter one was also found in *E. lathyris* seeds (contig TRINITY_DN26754_c0_g1_i4, 100% identity) and *E. tirucalli* leaves and stems (92% identity). One OSC from *E. lathyris* seeds (contig TRINITY_DN32011_c0_g1_i15) displayed 83% identity with a β-amyrin synthase from *E. tirucalli*. All sources of sequence information are indicated as uppercase numbers.

[1] Paired-end reads of *Euphorbia tirucalli* latex mRNA (SRR3407089) and EST sequences from *E. tirucalli* stems (HX984397-HX993567) were de novo assembled with *E. tirucalli* cDNA sequences obtained from the nucleotide and EST databases of National Center for Biotechnology Information. https://www.ncbi.nlm.nih.gov/nuccore/GETW01000000.

[2] *Euphorbia tirucalli* ; /db_xref="taxon:142860" ; /tissue_type="mixture of leaves and young stems" (Reference: Kajikawa M, Yamato KT, Fukuzawa H, Sakai Y, Uchida H, Ohyama K (2005) Cloning and characterization of a cDNA encoding beta-amyrin synthase from petroleum plant *Euphorbia tirucalli* L. Phytochemistry 66: 1759-1766).

3 *Euphorbia lathyris*_Mseed (reference: Luo D, Callari R, Hamberger B, Wubshet SG, Nielsen MT, Andersen-Ranberg J, Hallstrom BM, Cozzi F, Heider H, Lindberg Møller B, Staerk D, Hamberger B (2016) Oxidation andcyclization of casbene in the biosynthesis of Euphorbia factors from mature seeds of *Euphorbia lathyris* L. Proc Natl Acad Sci USA 113: E5082-9) Transcriptome data from https://www.ebi.ac.uk/ena/data/view/PRJNA282739.

Figure S6. Phylogenetic tree positioning the *Euphorbia lathyris* and *Euphorbia tirucalli* OSC protein sequences into the cyclase groups already characterized biochemically, here noted non steroidal (ns or steroidal (s) triterpene synthases (TTS).

List of characterised cyclases was obtained from Gas-Pascual et al. [58]. The phylogenetic method is based on MUSCLE alignment, gBLOCK alignment correction, PHYLIP for maximum likelihood method inference and Treedyn for graphical representation. Sequences from *Euphorbia lathyris* and *Euphorbia tirucalli* are highlighted in red. sTTS: steroidal triterpene synthase; nsTTS: steroidal triterpene synthase; CAS: cycloartenol synthase; LAS: lanosterol synthase; The accession number are the following: AAD05032: mixed synthase [Arabidopsis thaliana]; AAB94341: mixed synthase [Arabidopsis thaliana]; BAG82628: beta-amyrin synthase [Arabidopsis thaliana]; AAO33580: mixed synthase [Lotus japonicus]; BAA89815: beta-amyrin synthase [Glycirrhiza glabra]; BAE53429: beta-amyrin synthase [Lotus japonicus]; CAD23247: beta-amyrin synthase [Medicago truncatula]; BAA97558: beta-amyrin synthase [Pisum sativum]; BAA97559: mixed synthase [Pisum sativum]; BAB83088: beta-amyrin synthase [Betula platyphylla]; BAA33461: beta-amyrin synthase [Panax ginseng]; BAA33722: beta-amyrin synthase [Panax ginseng]; BAE43642: beta-amyrin synthase [Euphorbia tirucalli]; ADU52574: beta-amyrin synthase [Solanum lycopersicum]; ADU52575: mixed synthase [Solanum lycopersicum]; ACO24697: beta-amyrin synthase [Gentiana straminea]; BAB68529: Isomultiflorenol [Luffa cylindrica]; BAB83087: lupeol synthase [Betula platyphylla]; BAD08587: lupeol synthase [Glycirrhiza glabra]; BAE53430: lupeol

synthase [Lotus japonicus]; BAA86930: lupeol synthase [Olea europaea]; BAA86932: lupeol synthase [Taraxacum officinale]; CAC84558: beta-amyrin synthase [Avena strigosa]; AAA16975: lanosterol synthase [Saccharomyces cerevisiae]; NP_869736: cycloartenol synthase [Rhodopirellula baltica SH 1]; CBN75619: cycloartenol synthase [Ectocarpus siliculosu]; CAD39196: cycloartenol synthase [Stigmatella aurantiaca DW4/3-1]; EGD81137: cycloartenol synthase [Salpingoeca sp. ATCC 50818]; AAF80384: cycloartenol synthase [Dictyostelium discoideum]; EFA81207: cycloartenol synthase [Polysphondylium pallidum PN500]; EGG18936: cycloartenol synthase [Dictyostelium fasciculatum]; EFC50118: cycloartenol synthase [Naegleria gruber]; CCO17148: cycloartenol synthase [Bathycoccus prasino]; CAL52737: cycloartenol synthase [Ostreococcus tauri]; EDP09612: cycloartenol synthase [Chlamydomonas reindhartii]; EIE20900: cycloartenol synthase [Coccomyxa subellipsoidea C-169]; BAD34645: cucurbitadienol synthase [Cucurbita pepo]; BAI48072: cycloartenol synthase [Polypodiodes niponica]; BAF93208: cycloartenol synthase [Adiantus capitis-veneris]; AAG44096: cycloartenol synthase [Abies magnifica]; BAA84603: cycloartenol synthase [Allium macrosternom]; CAC84559: cycloartenol synthase [Avena strigosa]; AAT38892: cycloartenol synthase [Avena ventricosa]; BAH00370: cycloartenol synthase [Oryza sativa]; BAB83253: cycloartenol synthase [Costus speciosus]; BAB83254: mixed synthase [Costus speciosus]; BAB83085: cycloartenol synthase [Betula platyphylla]; BAD34644: cycloartenol synthase [Cucurbita pepo]; BAA85266: cycloartenol synthase [Luffa cylindrica]; NP_001233784: cycloartenol synthase [Solanum lycopersicum]; BAA33460: cycloartenol synthase [Panax ginseng]; BAB83086: cycloartenol synthase [Betula platyphylla]; BAA76902: cycloartenol synthase [Glycirrhiza glabra]; BAE53431: cycloartenol synthase [Lotus japonicus]; BAA23533: cycloartenol synthase [Pisum sativum]; AAC04931: cycloartenol synthase [Arabidopsis thaliana]; BAF73930: cycloartenol synthase [Kandelia candel]; BAF73929: cycloartenol synthase [Rhizophora stylosa]; BAE95408: lanosterol synthase [Arabidopsis thaliana]; BAA33462: lanosterol synthase [Panax ginseng]; BAE95410: lanosterol synthase [Lotus japonicus]; XP_001266126: oxidosqualene:lanosterol cyclase [Aspergillus fischeri NRRL 181]; XP_001262649: squalene-hopene-cyclase, putative [Aspergillus fischeri NRRL 181]; BAF93209: hydroxyhopane synthase [Adiantum capillus-veneris]; CAA51958: Squalene Hopene Cyclase [Zymomonas mobilis]; AFZ93646: cycloartenol synthase, partial [Euphorbia lathyris]; BAE43643: unknown function [Euphorbia tirucalli]; contig104_seq1: CAS1 [Euphorbia lathyris]; contig104_seq4: BUT1 [Euphorbia lathyris]; contig848_seq1: LAS1 [Euphorbia lathyris]; TRINITY_DN32011_c0_g1_i15: unknown function [Euphorbia lathyris]; TRINITY_DN26754_c0_g1_i4 unknown function

[Euphorbia lathyris]; GETW01011235_GETW01011235.1 unknown function [Euphorbia tirucalli]; GETW01001947_GETW01001947.1 unknown function [Euphorbia tirucalli]; GETW01001556_GETW01001556.1 unknown function [Euphorbia tirucalli]; GETW01003768_GETW01003768.1 unknown function [Euphorbia tirucalli].

Figure S7. Chromatograms (TIC, GC-MS) showing the profile of crude unsaponifiable hexanic extracts of the ergosterol-auxotrophic yeast *erg7*. A, *erg7*. **B**, *erg7::ElCAS1* shown as an example of transgenic yeast expressing one of the various triterpene synthases. Nomenclature: 8, 2,3-oxidosqualene; 9, ergosterol; 10, 24-methylene pollinastanol; 11, 31-nor cycloartenol; 12, pollinastanol; III, cycloartenol. Nomenclature and mass spectra as in Table S1. Peaks that are not numbered are not terpenoids.

Figure S8. Triterpene analysis of the yeast *erg7* transformed with OSCs from *Euphorbia lathyris*. Yeasts were grown with D-[1-^{13}C]-galactose as the sole carbon source in the medium to induce the expression of *OSCs*. The isotopic enrichment (%) of neosynthesized products was calculated, based on abundance values of m/z ions and relative to values obtained from yeasts grown on [1-^{12}C]-galactose. **A**, *erg7::ElLAS1*, an enrichment of about 8.5 % for lanosterol and of 5.4 % for ergosterol was obtained; **B**, *erg7::ElBUT1*,the enrichment was 9.4 % for butyrospermol while ergosterol had the expected natural abundance value of ≈1%. **C**, mass spectrum of ^{13}C-enriched butyrospermol. Data represent the averages of three characteristic ions on one biological sample; error bars indicate the standard deviation from the mean. The number of stars above the bars specifies a significant different percentage values (P < 0.05, F-test, T-test and one-way ANOVA).

Figure S9. GC-based quantification of *Nicotiana benthamiana* leaf triterpene purified extracts after *in folia* agroinfiltration of P19 +HMGR + BUT1 (*Niben::BUT1-HMGR*) and P19 + HMGR (*Niben::HMGR*). Data represent the averages of three biological replicates; error bars indicate the standard deviation from the mean. The number of stars above the bars specifies a significant different quantitative value (P < 0.05, F-test and T-test). Each color represents an unrelated statistical group.

Figure S10. NMR spectra of butyrospermol. Butyrospermyl acetate purified from *Euphorbia lathyris* latex (RMN2) or from *Nicotiana benthamiana* (RMN4) gave the following identical spectra (^{1}H NMR, 500MHz, $CDCl_3$): δ 5.23 (m, $1H_7$), 5.08 (t, $1H_{24}$, J=7.02 Hz), 4.51 (dd, $1H_3$,

J=11.3 Hz, J=4.0 Hz), 2.03 (s, 3H acetate), 1.67 (s, $3H_{30}$), 1.59 (s, $3H_{29}$), 0.95 (s, 3H), 0.91 (s, 3H), 0.83 (d, 3H, J=6.3Hz), 0.83 (s, 3H), 0.78 (s, 3H), 0.74 (s, 3H).

Supplemental tables

Table S1. Nomenclature and mass spectral data (EI 70 eV) of compounds relevant to this work (as acetate derivatives). The table follows an operational numbering of compounds: I-VI, main latex triterpenes; i-ii, minor latex triterpenes; 1-7, sterols and pentacyclic triterpenes; T1-T5, triterpene products formed in the yeast *erg7* expressiong various OSCs; t1-t8, triterpenes found in *Euphorbia lathyris* green parts.

Table S2. OSC accuracy assessment for *Euphorbia lathyris*. OSC accuracy was defined by the ratio of the primary product P_1 to the second most abundant product P_2 (or to the sum of all products) formed by a given OSC catalyst [45]. Values are calculated from the data sets shown in **Figure 4 and Figure 5.**

Table S3. Sterol biosynthetic genes in *Euphorbia lathyris*. BLAST analysis of the transcriptome data generated in the 'EULAFUEL' project was executed using *Arabidopsis thaliana* AGI (characterized plant sterol biosynthetic genes). Enzymes implied in the conversion of squalene into sterol pathway-end products (sitosterol, stigmasterol) and their acyl or glucosyl conjugates are listed in biosynthetic order.

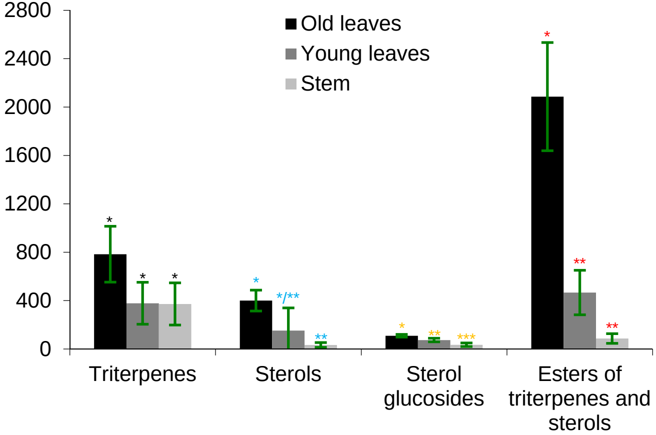

2800
2400
2000
1600
1200
800
400
0
Old leaves
Young leaves
Stem
*
*/**
**
***
Triterpenes
Sterols
Sterol glucosides
Esters of triterpenes and sterols

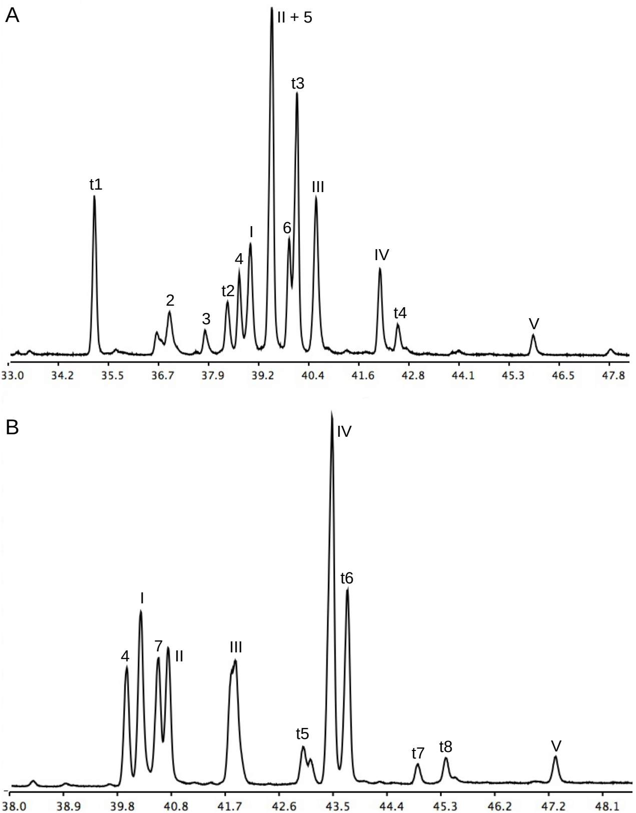
A
II + 5
t3
t1
III
I
6
4
IV
t2
2
t4
3
V
33.0
34.2
35.5
36.7
37.9
39.2
40.4
41.6
42.8
44.1
45.3
46.5
47.8
B
IV
t6
I
7
4
II
III
t5
t7
t8
V
38.0
38.9
39.8
40.8
41.7
42.6
43.5
44.4
45.3
46.2
47.2
48.1

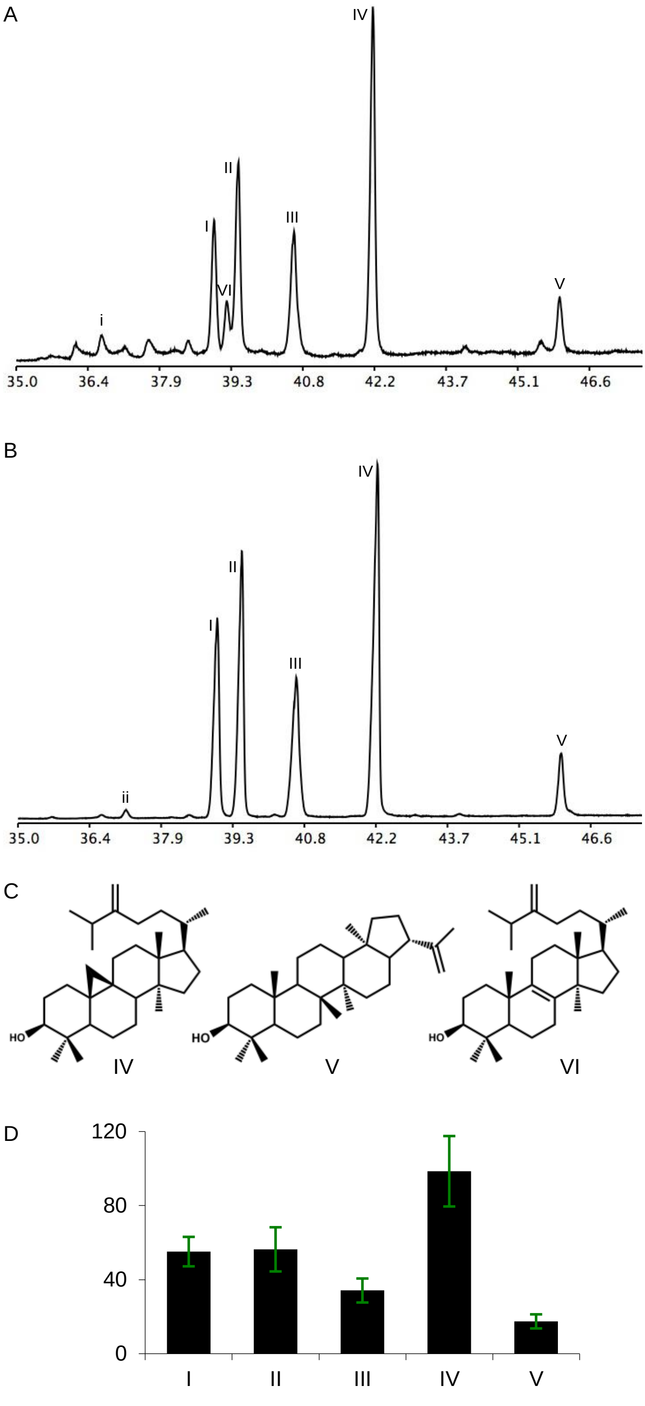
A
IV
II
I
III
VI
V
i
35.0
36.4
37.9
39.3
40.8
42.2
43.7
45.1
46.6
B
IV
II
I
III
V
ii
35.0
36.4
37.9
39.3
40.8
42.2
43.7
45.1
46.6
C
HO
IV
HO
V
HO
VI
D
120
80
40
0
I
II
III
IV
V

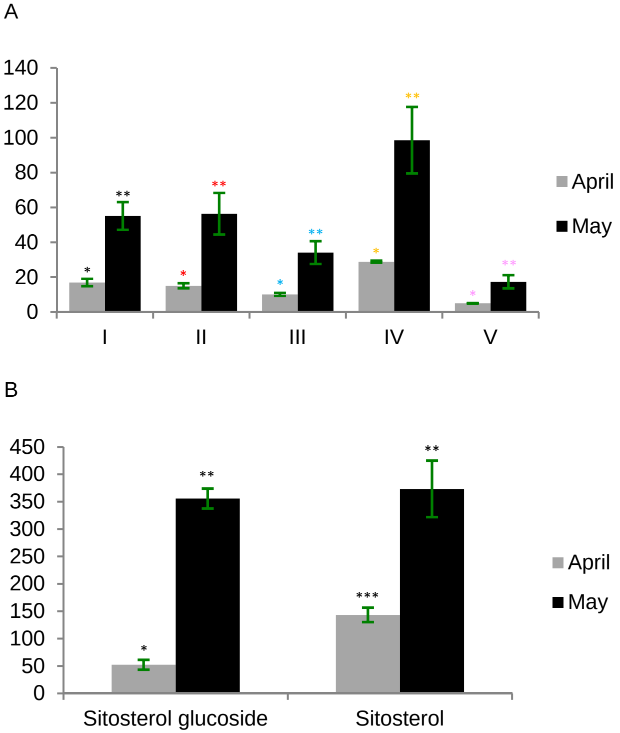
A
140
120
100
80
60
40
20
0
I
II
III
IV
V
April
May
B
450
400
350
300
250
200
150
100
50
0
Sitosterol glucoside
Sitosterol
April
May

Contig Name	Evidence	Specie	Source	Assembly or Methods	AA length	GETW01001556_GETW01001556.	contig104_seq4	BAE43642.1	GETW01001947_GETW01001947.	TRINITY_DN32011_c0_g1_i15	BAE43643.1	GETW01011235_GETW01011235.	contig848_seq1	GETW01003768_GETW01003768.	contig104_seq1	TRINITY_DN26754_c0_g1_i4
GETW01001556_GETW01001556.1		*E. tirucalli*	latex + stem + cDNAs [1]	MIRA+Trinity+EviGene	608	100	75,62	58,18	54,71	55,21	54,88	54,88	52,81	51,32	51,49	51,49
contig104_seq4	BUT1 (our work)	*E. lathyris*	latex (our study)	ABySS and Velvet	761	75,62	100	55,41	51,72	52,52	50,92	50,92	48,88	50,63	50,07	50,07
BAE43642.1	B-amyrin synth.	*E. tirucalli*	mixture of leaves and young stems [2]	Race PCR	762	58,18	55,41	100	70,83	73,27	71,62	71,62	59,42	58,87	58,5	58,5
GETW01001947_GETW01001947.1		*E. tirucalli*	latex + stem + cDNAs [1]	MIRA+Trinity+EviGene	763	54,71	51,72	70,83	100	80,66	78,98	78,98	55,13	56,27	55,79	55,79
TRINITY_DN32011_c0_g1_i15		*E. lathyris*	seeds [3]	Trinity+EviGene	637	55,21	52,52	73,27	80,66	100	83,49	83,49	55,12	56,38	57,17	57,17
BAE43643.1		*E. tirucalli*	mixture of leaves and young stems [2]	Race PCR	766	54,88	50,92	71,62	78,98	83,49	100	100	53,29	56,2	55,6	55,6
GETW01011235_GETW01011235.1		*E. tirucalli*	latex + stem + cDNAs [1]	MIRA+Trinity+EviGene	794	54,88	50,92	71,62	78,98	83,49	100	100	53,29	56,2	55,6	55,6
contig848_seq1	LAS1 (our work)	*E. lathyris*	latex (our study)	ABySS and Velvet	794	52,81	48,88	59,42	55,13	55,12	53,29	53,29	100	68,97	66,4	66,4
GETW01003768_GETW01003768.1		*E. tirucalli*	latex + stem + cDNAs [1]	MIRA+Trinity+EviGene	639	51,32	50,63	58,87	56,27	56,38	56,2	56,2	68,97	100	92,01	92,01
contig104_seq1	CAS1 (our work)	*E. lathyris*	latex (our study)	ABySS and Velvet	760	51,49	50,07	58,5	55,79	57,17	55,6	55,6	66,4	92,01	100	100
TRINITY_DN26754_c0_g1_i4		*E. lathyris*	seeds [3]	Trinity+EviGene	761	51,49	50,07	58,5	55,79	57,17	55,6	55,6	66,4	92,01	100	100

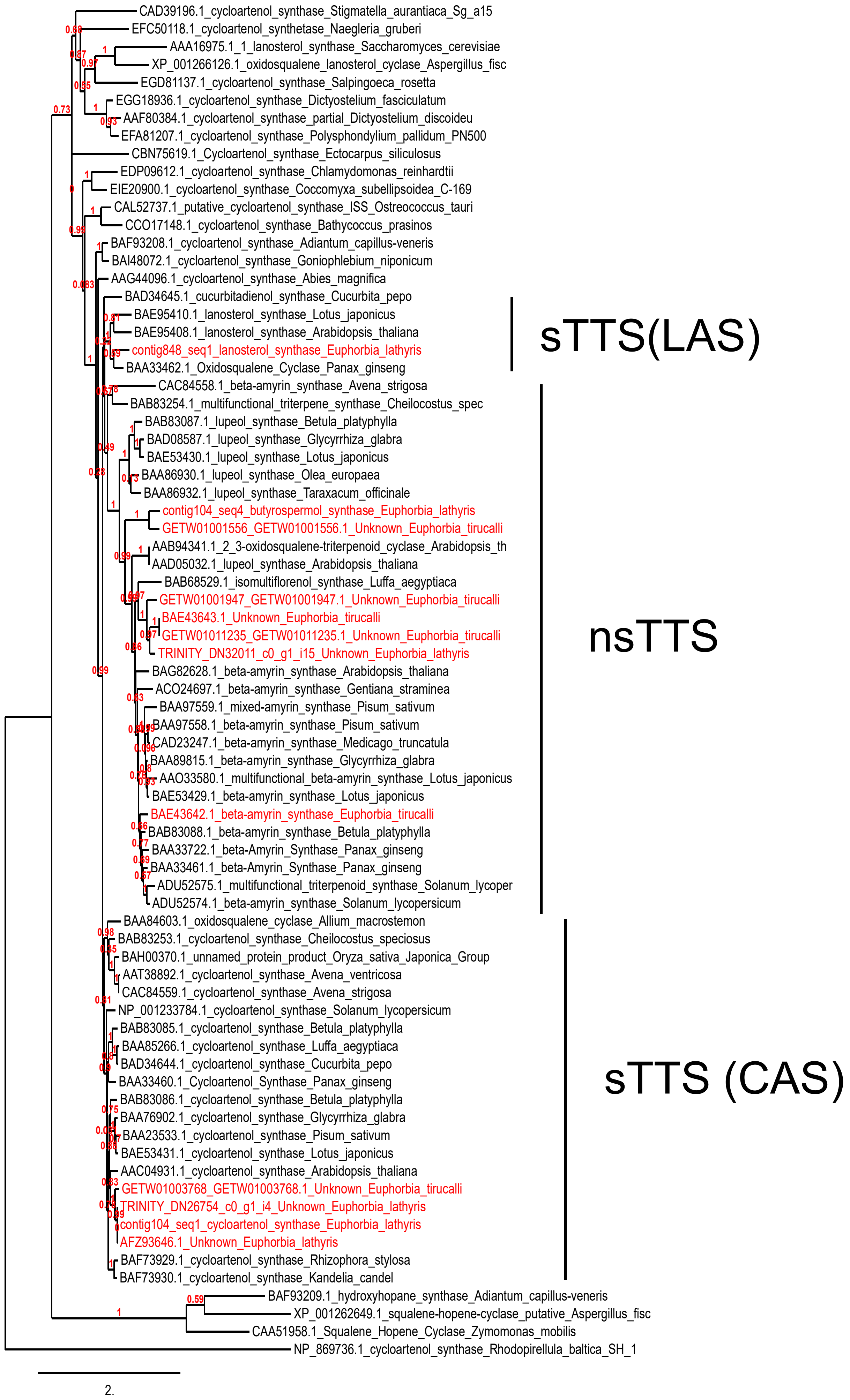
CAD39196.1_cycloartenol_synthase_Stigmatella_aurantiaca_Sg_a15
EFC50118.1_cycloartenol_synthetase_Naegleria_gruberi
AAA16975.1_1_lanosterol_synthase_Saccharomyces_cerevisiae
XP_001266126.1_oxidosqualene_lanosterol_cyclase_Aspergillus_fisc
EGD81137.1_cycloartenol_synthase_Salpingoeca_rosetta
EGG18936.1_cycloartenol_synthase_Dictyostelium_fasciculatum
AAF80384.1_cycloartenol_synthase_partial_Dictyostelium_discoideu
EFA81207.1_cycloartenol_synthase_Polysphondylium_pallidum_PN500
CBN75619.1_Cycloartenol_synthase_Ectocarpus_siliculosus
EDP09612.1_cycloartenol_synthase_Chlamydomonas_reinhardtii
EIE20900.1_cycloartenol_synthase_Coccomyxa_subellipsoidea_C-169
CAL52737.1_putative_cycloartenol_synthase_ISS_Ostreococcus_tauri
CCO17148.1_cycloartenol_synthase_Bathycoccus_prasinos
BAF93208.1_cycloartenol_synthase_Adiantum_capillus-veneris
BAI48072.1_cycloartenol_synthase_Goniophlebium_niponicum
AAG44096.1_cycloartenol_synthase_Abies_magnifica
BAD34645.1_cucurbitadienol_synthase_Cucurbita_pepo
BAE95410.1_lanosterol_synthase_Lotus_japonicus
BAE95408.1_lanosterol_synthase_Arabidopsis_thaliana
contig848_seq1_lanosterol_synthase_Euphorbia_lathyris
BAA33462.1_Oxidosqualene_Cyclase_Panax_ginseng
sTTS(LAS)
CAC84558.1_beta-amyrin_synthase_Avena_strigosa
BAB83254.1_multifunctional_triterpene_synthase_Cheilocostus_spec
BAB83087.1_lupeol_synthase_Betula_platyphylla
BAD08587.1_lupeol_synthase_Glycyrrhiza_glabra
BAE53430.1_lupeol_synthase_Lotus_japonicus
BAA86930.1_lupeol_synthase_Olea_europaea
BAA86932.1_lupeol_synthase_Taraxacum_officinale
contig104_seq4_butyrospermol_synthase_Euphorbia_lathyris
GETW01001556_GETW01001556.1_Unknown_Euphorbia_tirucalli
AAB94341.1_2_3-oxidosqualene-triterpenoid_cyclase_Arabidopsis_th
AAD05032.1_lupeol_synthase_Arabidopsis_thaliana
BAB68529.1_isomultiflorenol_synthase_Luffa_aegyptiaca
GETW01001947_GETW01001947.1_Unknown_Euphorbia_tirucalli
BAE43643.1_Unknown_Euphorbia_tirucalli
GETW01011235_GETW01011235.1_Unknown_Euphorbia_tirucalli
TRINITY_DN32011_c0_g1_i15_Unknown_Euphorbia_lathyris
BAG82628.1_beta-amyrin_synthase_Arabidopsis_thaliana
ACO24697.1_beta-amyrin_synthase_Gentiana_straminea
BAA97559.1_mixed-amyrin_synthase_Pisum_sativum
BAA97558.1_beta-amyrin_synthase_Pisum_sativum
CAD23247.1_beta-amyrin_synthase_Medicago_truncatula
BAA89815.1_beta-amyrin_synthase_Glycyrrhiza_glabra
AAO33580.1_multifunctional_beta-amyrin_synthase_Lotus_japonicus
BAE53429.1_beta-amyrin_synthase_Lotus_japonicus
BAE43642.1_beta-amyrin_synthase_Euphorbia_tirucalli
BAB83088.1_beta-amyrin_synthase_Betula_platyphylla
BAA33722.1_beta-Amyrin_Synthase_Panax_ginseng
BAA33461.1_beta-Amyrin_Synthase_Panax_ginseng
ADU52575.1_multifunctional_triterpenoid_synthase_Solanum_lycoper
ADU52574.1_beta-amyrin_synthase_Solanum_lycopersicum
nsTTS
BAA84603.1_oxidosqualene_cyclase_Allium_macrostemon
BAB83253.1_cycloartenol_synthase_Cheilocostus_speciosus
BAH00370.1_unnamed_protein_product_Oryza_sativa_Japonica_Group
AAT38892.1_cycloartenol_synthase_Avena_ventricosa
CAC84559.1_cycloartenol_synthase_Avena_strigosa
NP_001233784.1_cycloartenol_synthase_Solanum_lycopersicum
BAB83085.1_cycloartenol_synthase_Betula_platyphylla
BAA85266.1_cycloartenol_synthase_Luffa_aegyptiaca
BAD34644.1_cycloartenol_synthase_Cucurbita_pepo
BAA33460.1_Cycloartenol_Synthase_Panax_ginseng
BAB83086.1_cycloartenol_synthase_Betula_platyphylla
BAA76902.1_cycloartenol_synthase_Glycyrrhiza_glabra
BAA23533.1_cycloartenol_synthase_Pisum_sativum
BAE53431.1_cycloartenol_synthase_Lotus_japonicus
AAC04931.1_cycloartenol_synthase_Arabidopsis_thaliana
GETW01003768_GETW01003768.1_Unknown_Euphorbia_tirucalli
TRINITY_DN26754_c0_g1_i4_Unknown_Euphorbia_lathyris
contig104_seq1_cycloartenol_synthase_Euphorbia_lathyris
AFZ93646.1_Unknown_Euphorbia_lathyris
BAF73929.1_cycloartenol_synthase_Rhizophora_stylosa
BAF73930.1_cycloartenol_synthase_Kandelia_candel
sTTS (CAS)
BAF93209.1_hydroxyhopane_synthase_Adiantum_capillus-veneris
XP_001262649.1_squalene-hopene-cyclase_putative_Aspergillus_fisc
CAA51958.1_Squalene_Hopene_Cyclase_Zymomonas_mobilis
NP_869736.1_cycloartenol_synthase_Rhodopirellula_baltica_SH_1
2.

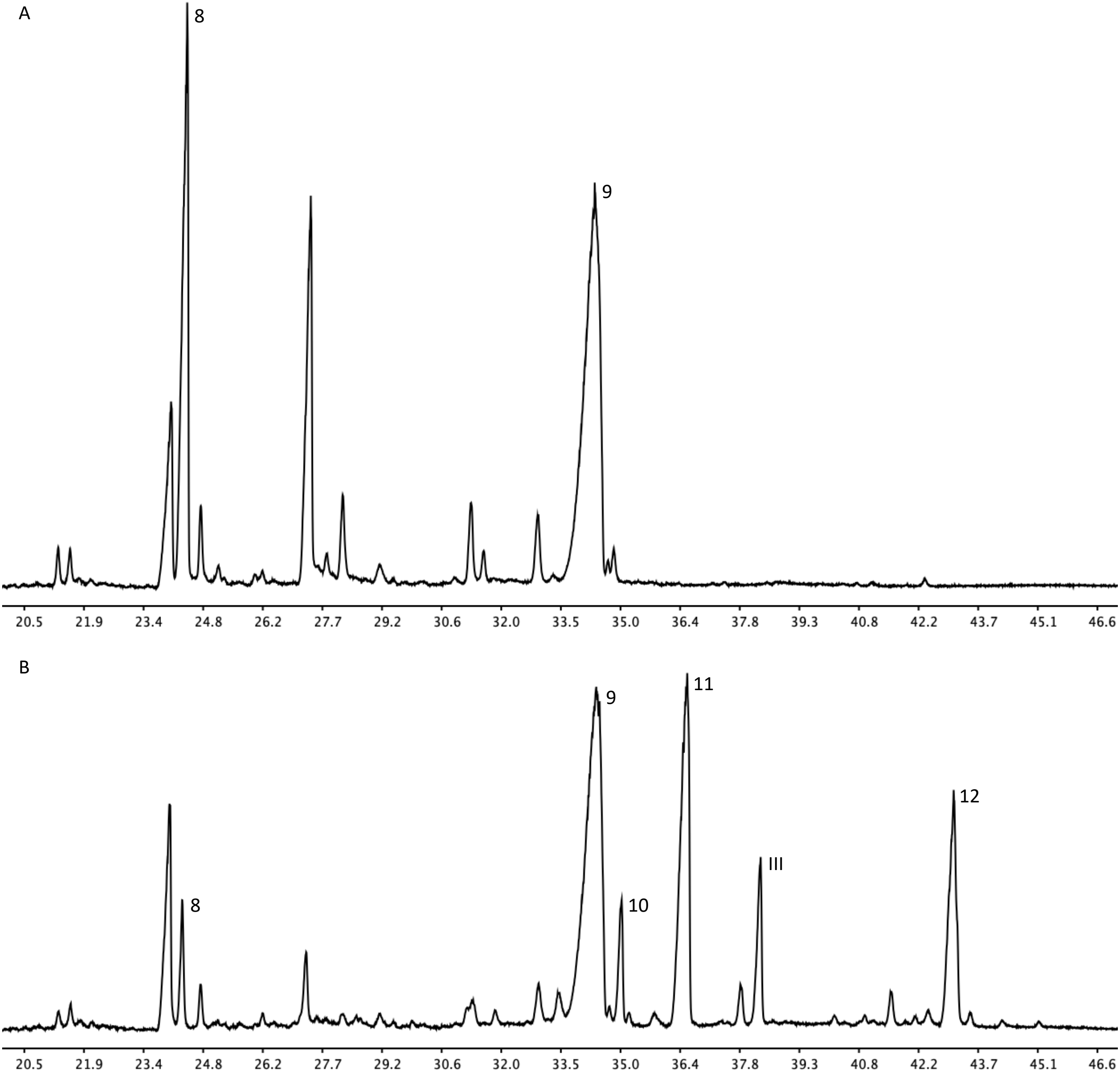
A
8
9
20.5
21.9
23.4
24.8
26.2
27.7
29.2
30.6
32.0
33.5
35.0
36.4
37.8
39.3
40.8
42.2
43.7
45.1
46.6
B
8
9
10
11
III
12
20.5
21.9
23.4
24.8
26.2
27.7
29.2
30.6
32.0
33.5
35.0
36.4
37.8
39.3
40.8
42.2
43.7
45.1
46.6

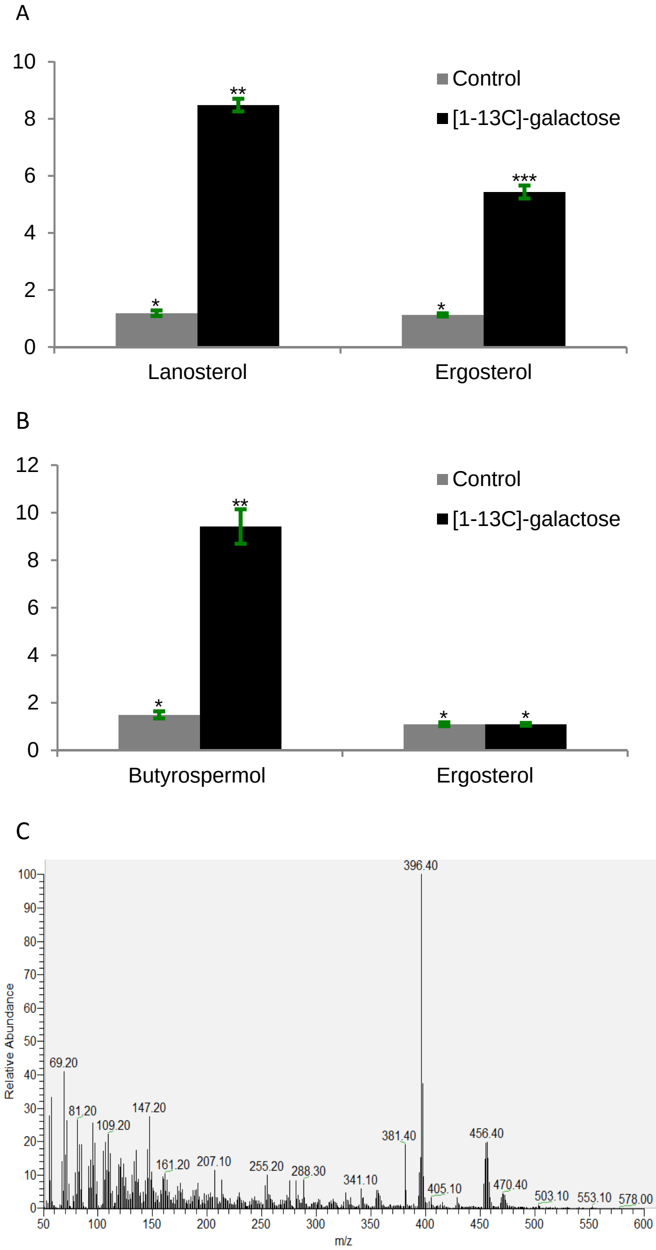

A
10
8
6
4
2
0
Control
[1-13C]-galactose
**
***
*
*
Lanosterol
Ergosterol
B
12
10
8
6
4
2
0
Control
[1-13C]-galactose
**
*
*
*
Butyrospermol
Ergosterol
C
Relative Abundance
100
90
80
70
60
50
40
30
20
10
0
396.40
69.20
81.20
147.20
109.20
381.40
456.40
161.20
207.10
255.20
288.30
341.10
405.10
470.40
503.10
553.10
578.00
50
100
150
200
250
300
350
400
450
500
550
600
m/z

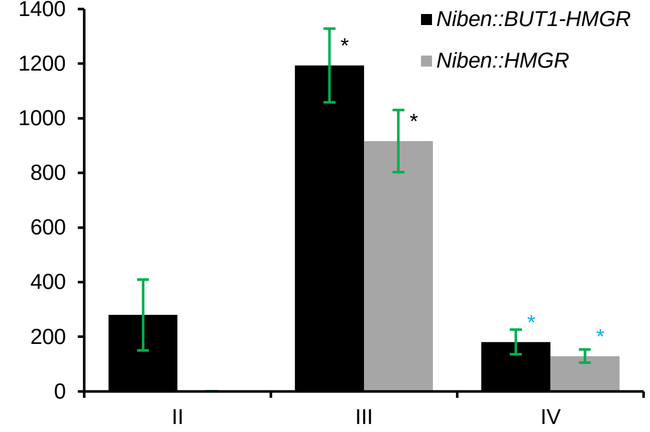

Niben::BUT1-HMGR
Niben::HMGR
1400
1200
1000
800
600
400
200
0
*
*
*
*
II
III
IV

RMN 2

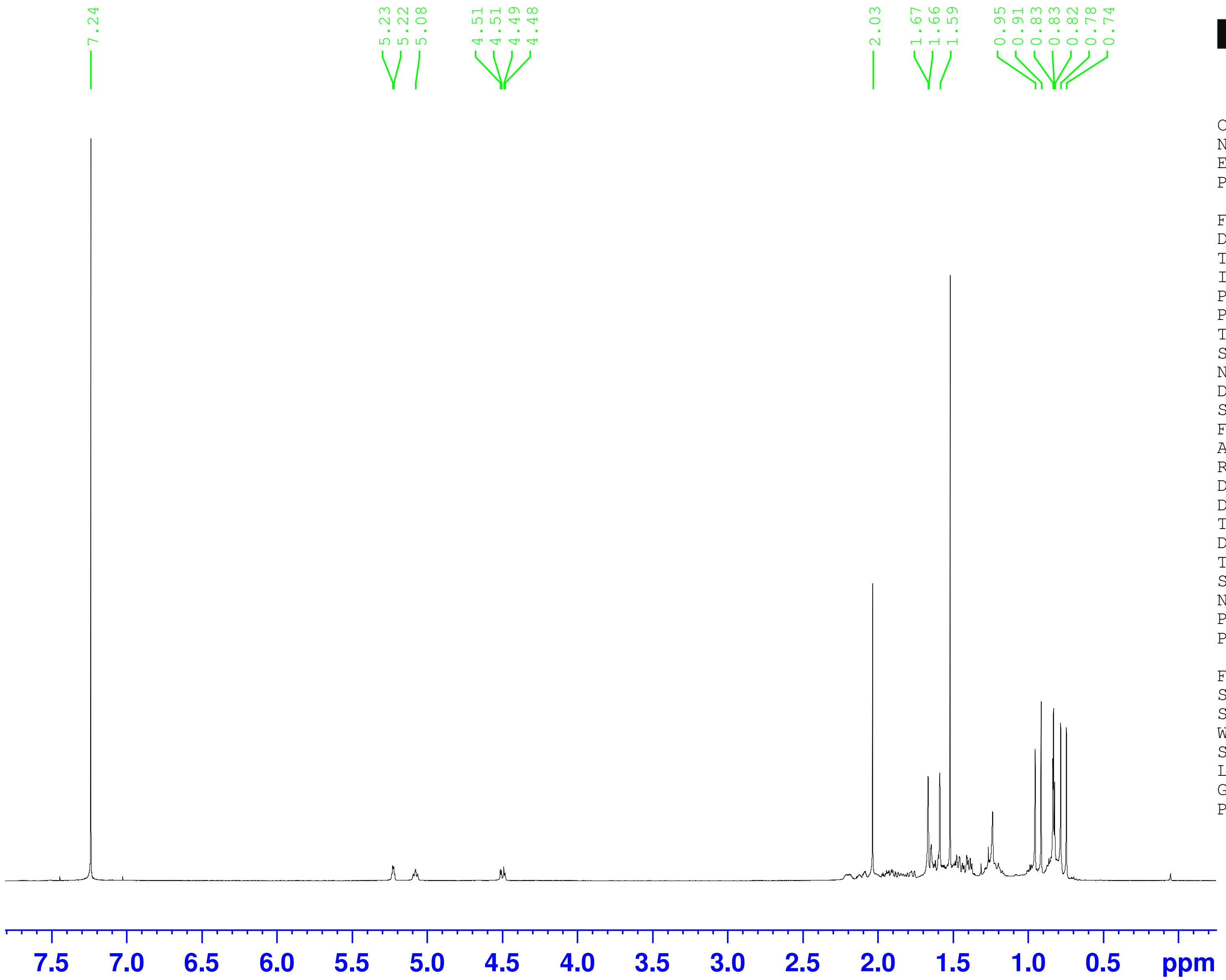

Current Data Parameters

NAME	IBMP-170427
EXPNO	20
PROCNO	1

F2 - Acquisition Parameters

Date_	20170427
Time	16.33 h
INSTRUM	spect
PROBHD	Z127784_0003 (
PULPROG	zg30
TD	69998
SOLVENT	CDCl3
NS	32
DS	2
SWH	10000.000 Hz
FIDRES	0.285722 Hz
AQ	3.4999001 sec
RG	40.3
DW	50.000 usec
DE	18.00 usec
TE	298.0 K
D1	0.50000000 sec
TD0	1
SFO1	500.2430014 MHz
NUC1	1H
P1	11.00 usec
PLW1	12.84000015 W

F2 - Processing parameters

SI	131072
SF	500.2400225 MHz
WDW	EM
SSB	0
LB	0.10 Hz
GB	0
PC	1.00

RMN 4

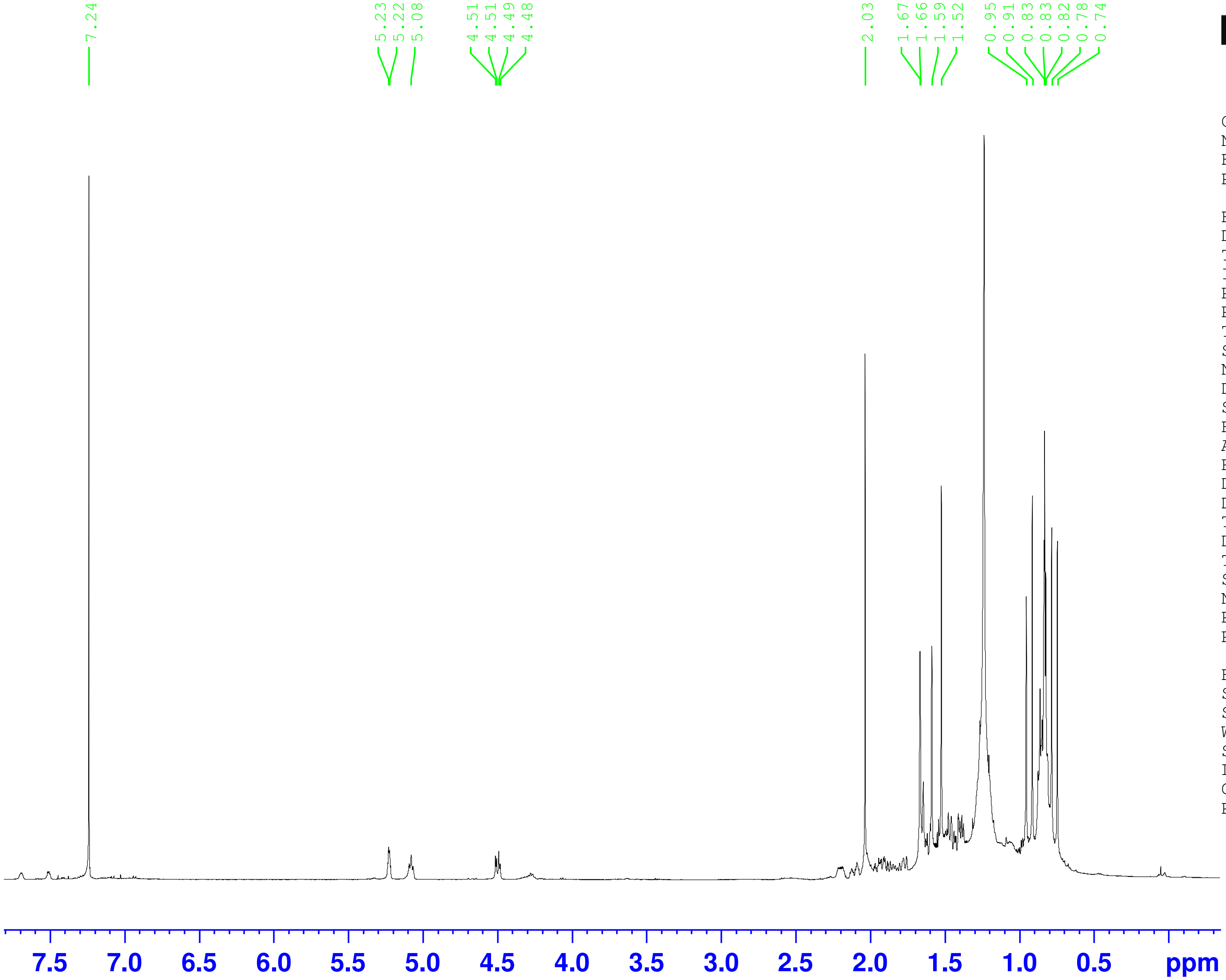

Current Data Parameters

NAME	IBMP-170427	
EXPNO	30	
PROCNO	1	

F2 - Acquisition Parameters

Date_	20170427	
Time	16.38	h
INSTRUM	spect	
PROBHD	Z127784_0003 (	
PULPROG	zg30	
TD	69998	
SOLVENT	CDCl3	
NS	32	
DS	2	
SWH	10000.000	Hz
FIDRES	0.285722	Hz
AQ	3.4999001	sec
RG	25.4	
DW	50.000	usec
DE	18.00	usec
TE	298.0	K
D1	0.50000000	sec
TD0	1	
SFO1	500.2430014	MHz
NUC1	1H	
P1	11.00	usec
PLW1	12.84000015	W

F2 - Processing parameters

SI	131072	
SF	500.2400224	MHz
WDW	EM	
SSB	0	
LB	0.20	Hz
GB	0	
PC	1.00	
